# Reducing frailty in frail people with multiple sclerosis: Feasibility of a 6-week multimodal exercise training program

**DOI:** 10.1371/journal.pone.0347063

**Published:** 2026-04-15

**Authors:** Tobia Zanotto, Abbas Tabatabaei, Sharon G. Lynch, Jianghua He, Madison Lysaught, Pedram Ahmadnezhad, Jane Ibude, Hannes Devos, Lee D. Chaves, Bruce R. Troen, Jacob J. Sosnoff

**Affiliations:** 1 Department of Occupational Therapy Education, School of Health Professions, University of Kansas Medical Center, Kansas City, Kansas, United States of America; 2 Landon Center on Aging, University of Kansas Medical Center, Kansas City, Kansas, United States of America; 3 Human Performance Laboratory, University of Kansas Medical Center, Kansas City, Kansas, United States of America; 4 Department of Physical Therapy, Rehabilitation Science, and Athletic Training, School of Health Professions, University of Kansas Medical Center, Kansas City, Kansas, United States of America; 5 School of Physical Therapy and Rehabilitation Sciences, University of South Florida, Tampa, Florida, United States of America; 6 Department of Neurology, School of Medicine, University of Kansas Medical Center, Kansas City, Kansas, United States of America; 7 Department of Biostatistics and Data Science, University of Kansas Medical Center, Kansas City, Kansas, United States of America; 8 School of Health & Human Sciences, Department of Physical Therapy, Indiana University, Indianapolis, Indiana, United States of America; 9 Division of Geriatrics, Department of Internal Medicine, School of Medicine, University of Kansas Medical Center, Kansas City, Kansas, United States of America; 10 Research Service, Kansas City Veterans Affairs Healthcare System, Kansas City, Missouri, United States of America; Tohoku Daigaku, JAPAN

## Abstract

**Background:**

Frailty is increasingly recognized as a prevalent and debilitating condition in people with multiple sclerosis (MS) and is linked to poorer health outcomes. However, targeted interventions remain limited. The objective of this study was to examine the feasibility of a multimodal exercise training (MET) program to reduce frailty in frail people with MS.

**Materials and methods:**

Sixteen frail people with MS (age = 55.0 ± 7.7 years, 81.3% female, Fried frailty score ≥3) participated in this pilot randomized controlled trial. Participants were randomly assigned to a 6-week MET program consisting of virtual reality treadmill training + resistance training (n = 8) or to a waitlist control group (n = 8). Feasibility outcomes included recruitment, retention, and adherence rates as well as safety and user engagement throughout the study (Study Participant Feedback Questionnaire—SPFQ). Exploratory outcomes were collected at baseline and 6 weeks and included the Evaluative Frailty Index for Physical Activity (EFIP), the 54-item MS Quality of Life questionnaire (MSQoL-54), the Modified Fatigue Impact Scale (MFIS), and the Physiological Profile Assessment (PPA).

**Results:**

Fourteen participants, eight in the intervention group and six in the control group, completed the study. The recruitment rate was 0.33 participants/week, retention was 87.5%, and adherence was high, with participants completing 97.2% of planned training sessions. No adverse events or training-related pain were recorded. The data collection procedures were successfully implemented with complete outcome data. Participants agreed or strongly agreed with 95.7% of applicable SPFQ items, indicating high levels of engagement and satisfaction with the trial. Between-group differences in baseline to 6-week change were: EFIP −0.07 (95% CI: −0.14, −0.00); MSQoL-54 mental health +21.24 (95% CI: 7.32, 35.16); MSQoL-54 physical health +19.26 (95% CI: 5.61, 32.91); MFIS −11.46 (95% CI: −18.34, −5.13); and PPA −0.09 (95% CI: −1.19, 1.01).

**Conclusion:**

The MET program was safe, feasible, and well-received by frail people with MS. These findings support the viability of MET for future larger-scale trials targeting frailty reduction in this population.

**Trial registration:**

ClinicalTrials.gov NCT06042244

## Introduction

Frailty is a syndrome of diminished reserve and resilience arising from the accumulation of deficits across multiple physiologic systems that leads to vulnerability to adverse health outcomes [1]. The estimated prevalence of frailty in middle-aged (40–65 years of age) people with MS (pwMS) is two to four times higher than in older adults (≥65 years of age) without MS [2]. In addition, frailty is associated with reduced quality of life [3], adverse clinical outcomes [2,4], and common MS-related signs and symptoms in pwMS, including impaired mobility, muscle weakness, fatigue, mental health issues, and cognitive dysfunction [5–9]. Overall, these findings strongly suggest that minimizing frailty could have a positive impact on the health of pwMS. Therefore, there is a critical need to identify and evaluate strategies for reducing frailty in the MS community.

Exercise-based rehabilitation can improve physiological function and hallmarks of frailty such as walking speed, muscle mass, and fatigue in pwMS [10]. Therefore, it may represent a viable strategy to counteract frailty in this population. In a recent study, we provided initial evidence that a 6-week treadmill training (TT) program with and without virtual reality (VR) augmentation resulted in a reduction of frailty levels (pooled mean difference in frailty index: 0.024; 95% CI: 0.010–0.038) in 83 pwMS [11]. Compared to TT alone, however, participants randomized to TT augmented by VR (TT + VR) had a greater improvement in the cognitive frailty index. Importantly, TT + VR targets both motor and cognitive function, which are both affected by MS [12,13] and impaired in frail individuals [14–16]. Although our previous investigation had several merits, it also had some drawbacks. Specifically, frailty was not one of the inclusion criteria, and, due to the secondary analysis research design, the intervention was not conceived to treat frailty as the primary objective. In addition, it did not include a resistance training (RT) component, which is considered an essential active ingredient of exercise programs aiming to reduce frailty in older adults [17–19].

Progressive RT promotes anabolic effects leading to gains in muscle strength and reducing the risk of geriatric problems, such as frailty, in both older adults and people with chronic diseases [20,21]. Studies involving RT in the context of MS have shown that it is safe, tolerable, and that it improves muscle strength as well as MS-related symptoms [22–28]. Previous research in other clinical populations suggests that combining RT with other exercise modalities may represent the best strategy for counteracting frailty [29]. In this respect, combining our previous intervention (TT + VR) with progressive RT may have a synergistic effect on MS-related frailty, as the TT + VR component would target motor and cognitive function while the RT component would counteract the muscle weakness component of frailty. To date, however, it is not known whether this type of multimodal exercise training (MET) is feasible in frail pwMS.

The current study aimed to address this knowledge gap and move the field forward by examining the feasibility of a MET program consisting of motor-cognitive rehabilitation and progressive RT to reduce frailty in frail pwMS. We hypothesized the retention rate, and the proportion of completed training sessions would be more than 80%. Additional feasibility outcomes comprised participant recruitment, participant safety, the appropriateness of data collection and training procedures, evaluation of resources, and measures of user engagement. The effects of MET on exploratory frailty-related outcomes were also examined.

## Materials and methods

### Study design, setting, and participants

This study used a pilot two-parallel group assessor-blinded randomized controlled trial (RCT) study design and was prospectively registered in ClinicalTrials.gov (registration number: NCT06042244). All the study procedures, including screening and recruitment, were conducted in the Landon Center on Aging at the University of Kansas Medical Center (KUMC) between November 20, 2023, and February 23, 2025. The inclusion criteria were: 1) confirmed diagnosis of MS by the treating neurologist; 2) age between 40 and 65 years; 3) ability to walk without bilateral support, 4) ability to comprehend and follow instructions in English; 5) frailty, defined as meeting ≥ 3 criteria of the Fried frailty phenotype [30]. The exclusion criteria were: 1) diagnosis of other clinically important neurological conditions (e.g., Parkinson’s disease, epilepsy); 2) presence of active psychiatric problems; 3) unstable cardiovascular conditions; 4) acute lower back or lower limb pain, rheumatic and/or other severe orthopedic problems which could interfere with RT; 5) inability to walk 10 meters unassisted; 6) severe cognitive impairment, defined as a MiniCog score < 3 [31]. The study conformed with the 1964 Declaration of Helsinki and its later amendments and received ethical approval by the KUMC Institutional Review Board (STUDY00149742). Participants provided written informed consent prior to participating.

#### Randomization, blinding, and study groups.

Participants were randomly assigned to one of the two study groups (i.e., MET or waitlist control group) after the baseline assessment using pre-established randomization codes devised by the biostatistician. The processes of sequence generation, allocation concealment, and group allocation were implemented in a blinded and independent manner, in agreement with the CONSORT guidelines [32,33].

### MET (intervention group)

All procedures pertaining to the MET program are fully detailed in the published protocol [34]. Here, we provide a synopsis of the main aspects. Participants randomized to MET trained three times per week for six consecutive weeks (maximum number of training visits = 18). Each training session consisted of 15 minutes of TT + VR [11], followed by 30 minutes of progressive evidence-based RT [18,27], followed by another 15 minutes of TT + VR. As part of TT + VR, participants walked at a comfortable pace on the treadmill wearing a safety harness while navigating a non-immersive VR environment projected on a TV screen. During the walk, participants received automated, real-time feedback from the VR software (GaitBetter, Ramat Gan, Israel) [35]. The system provided visual performance indicators on the screen (i.e., a green light to signal adequate performance and a red light to indicate inadequate responses) based on participants’ accuracy in obstacle avoidance and pathway navigation. Comfortable walking speed on the treadmill was assessed on the first day of training and was used to select the treadmill speed. The speed was initially set at 80% of the participants’ comfortable walking speed and was progressively increased during the 6-week training period, using standard procedures from our previous studies [11,35]. As part of RT, participants performed evidence-based exercises involving both the lower (i.e., seated leg press and knee extensions) and upper body (i.e., seated chest press and lat-pulldowns), as fully detailed in the published protocol [34]. These exercises were selected based on recommendations for RT in pwMS and frail individuals [20,27]. Participants performed two sets of 8–12 repetitions for each exercise with recovery periods of up to two minutes between sets. The exercises were executed at 30% of the one-repetition maximum (1-RM) during the first week of training and slowly progressed up to 80% of the 1-RM. A detailed description of the 1-RM calculation and RT progression methods is available in the published protocol [34].

### Waitlist control group

Participants assigned to the waitlist control group received their usual treatment during the 6-week study period. At the end of the study, namely upon completion of the final assessment visit, they were offered the opportunity to receive the exercise intervention if they wished so. Participants were asked to avoid participating in other exercise trials during the 6-week study period; however, we did not impose any changes in prescribed medications or in lifestyle factors.

### Feasibility outcomes

To examine the feasibility of our MET program, we evaluated the following feasibility outcomes, using guiding principles for feasibility studies [36]: 1) Participant recruitment, evaluated relative to the a priori target sample size specified in the published protocol (n = 24), as well as descriptively using the observed recruitment rate (participants/week) and the proportion of approached individuals who consented to participate; 2) retention rate, defined as the ratio between the number of participants completing the intervention and those enrolled at baseline; 3) participant safety, evaluated through the number of adverse events recorded throughout the study and training-related pain (numeric pain rating scale—NPRS [37]); 4) appropriateness of data collection procedures, evaluated through the percentage of missing data for analysis purposes; 5) appropriateness of the training procedures, defined as the time spent in training (i.e., adherence) and the number of training sessions completed by participants; 6) evaluation of resources, quantified as the difference between proposed and actual study timeline; 7) user engagement, evaluated using the Study Participant Feedback Questionnaire (SPFQ) [38,39].

### Exploratory outcomes

Exploratory outcomes were collected at baseline, prior to the randomization, and after the 6-week study period. Participants completed the Evaluative Frailty Index for Physical Activity (EFIP) [40] as the primary exploratory outcome. The EFIP is a frailty index based on the deficit accumulation model that was specifically designed to evaluate the effects of physical activity or exercise-based interventions on frailty [40]. This tool assesses 50 health-related items encompassing several frailty components, such as physical, psychological, general heath, and social functioning [40]. Previous research has demonstrated that EFIP is valid, reliable, and responsive to exercise-based interventions entailing motor-cognitive rehabilitation [41]. The secondary exploratory outcomes were the 54-item multiple sclerosis quality of life questionnaire (MSQoL-54), [42] the modified fatigue impact scale (MFIS), [43] and the Physiological Profile Assessment (PPA), [44] which were assessed as proxy measures of quality of life, fatigue, and physiological fall-risk (often seen as corollary measures of frailty), respectively. The MSQoL-54 has acceptable validity and reliability in pwMS [45]. This questionnaire provides an assessment of several domains of quality of life. Eight subscales with scores ranging from 0 to 100 are calculated, wherein higher scores indicate a higher quality of life. For this study, we took the ‘physical health’ (PHC) and ‘mental health’ composite (MHC) scores for the analysis. The MFIS is one of the most common patient-reported measure of fatigue in MS research [46]. This 21-item questionnaire enables the evaluation of four subscales of fatigue—i.e., physical, cognitive, psychological, and total fatigue. In this study, we took the total MFIS score for analysis purposes. The PPA has been widely used in pwMS [47,48] and assesses multiple physiological factors linked to fall-risk, such as vision (edge contrast sensitivity test), proprioception (lower-limb matching test with eyes closed), reaction time (simple reaction time test using a handheld electronic timer), balance (30-second postural sway test on a foam mat), and strength (isometric maximal knee extension strength). The results from each test are entered into the PPA software, which provides a Z-score representing the main outcome used for analysis. Notably, although EFIP and other outcomes were pre-specified as primary and secondary outcomes in the published protocol, [34] all effectiveness-related outcomes were treated as exploratory in the present manuscript due to the smaller-than-planned sample size and the feasibility focus of this pilot trial.

### Statistical methods

Statistical analyses were performed using SPSS (version 30.0 for Windows; IBM, Inc.). Descriptive statistics (frequencies and percentages for categorical variables and mean and standard deviation for continuous variables) were used to summarize the participants’ characteristics. While the protocol specified hypothesis-driven inferential analyses, the final sample size was substantially smaller than planned. Therefore, to avoid overinterpretation, statistical analyses were limited to descriptive summaries of within-group changes (means and standard deviations) and between-group differences (means and 95% confidence intervals). In addition, standardized effect size estimates (Cohen’s d) were calculated for between-group differences in baseline to 6-week change in exploratory outcomes to aid interpretation of the magnitude of observed effects. These estimates are presented descriptively only, as formal hypothesis testing was not performed, consistent with recommendations for pilot feasibility studies [49].

## Results

### Feasibility outcomes

#### Participant recruitment.

**Fig 1:**
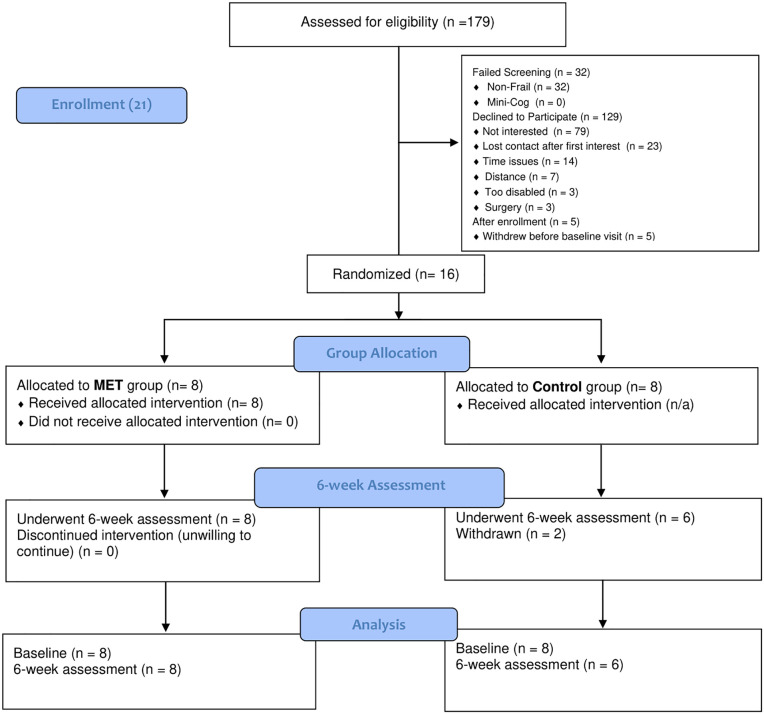
CONSORT flowchart. **Legend:** MET: multimodal exercise training.

Recruitment was conducted between December 5, 2023, and December 12, 2024. Fig 1 details the participant flow diagram throughout the study. Sixteen participants (8.9% of patients approached for consent; observed recruitment rate = 0.33 participants/week), corresponding to 67% of the a priori recruitment target (n = 24), completed the baseline assessment and were successfully randomized to MET (n = 8) or to the waitlist control group (n = 8). The baseline characteristics of the study participants are summarized in Table 1.

**Table 1:** Baseline participant characteristics: results are expressed as frequencies and percentages for categorical variables and mean ± standard deviation for continuous variables.

Variables	All participants (16)	Control (8)	MET (8)	p-value
Female Gender, n (%)	13 (81.3)	7 (87.5)	6 (75)	1.000
Age (years)	55.0 ± 7.7	57.6 ± 7.7	52.4 ± 7.3	0.195
BMI (kg/m^2^)	29.8 ± 8.1	31.2 ± 7.1	28.4 ± 9.2	0.574
Race				
White	10 (62.5)	4 (50.0)	6 (75.0)	0.608
Black	5 (31.3)	4 (50.0)	1 (12.5)	0.282
Hispanic	1 (6.3)	0 (0.0)	1 (12.5)	1.000
Type of MS				
RRMS	10 (62.5)	6 (75.0)	4 (50.0)	0.608
PPMS	3 (18.8)	2 (25.0)	1 (12.5)	1.000
SPMS	3 (18.8)	0 (0.0)	3 (37.5)	0.200
PDDS (score)	3.3 ± 1.0	3.3 ± 1.3	3.3 ± 0.8	0.902
EFIP (score)	0.31 ± 0.08	0.34 ± 0.08	0.27 ± 0.06	0.083

**Abbreviations:** BMI: body mass index; EFIP: evaluative frailty index for physical activity; MET: multimodal exercise training; PDDS: patient determined disease steps; PPMS: primary progressive MS; RRMS: relapsing remitting MS; SPMS: secondary progressive MS.

#### Participant retention.

Fourteen participants, eight in the intervention group and six in the control group, completed the study (overall retention rate = 87.5%). Two participants in the control group dropped out from the study due to medical complications unrelated to the study (n = 1) or loss to follow-up (n = 1). Both participants had relapsing–remitting MS, identified as Black, were over 50 years of age, and represented one male and one female.

#### Participant safety.

In terms of safety, no adverse events were recorded throughout the study and the median training-related pain (NPRS) score in the MET group was 0.0 (interquartile range = 0.0). In addition, none of the training sessions were stopped because of patient-reported pain or discomfort.

### Appropriateness of data collection and training procedures

During the assessment visits, no missing data in the primary or secondary exploratory outcomes occurred, suggesting that the data collection procedures were adequate. Regarding adherence, the eight participants in the MET group completed 140 out of the 144—i.e., 97.2%—planned training sessions, and the minimum number of sessions completed by a participant was 16 out of 18. Participants spent, on average, one hour in training during each session, except for two participants who were not able to complete the planned 30 minutes of treadmill walking due to fatigue and foot drop. Those two participants walked on average 18.8 and 19.6 minutes/session, respectively. In addition, all participants were able to perform the RT exercises and progress with the intensity of both treadmill training and RT, as detailed in the study protocol.

### Evaluation of resources

In terms of resources, the study was conducted from December 2023 (date of first enrollment) to February 2025 (date of last assessment), which aligned with the proposed project timeline at funding acquisition. However, the final sample size for data analysis was 30% lower than proposed in the published protocol (i.e., 10 participants in each group).

### User engagement

Participants agreed or strongly agreed on 95.7% of the 20 applicable SPFQ items, indicating high levels of engagement and satisfaction with the trial. The SPFQ item with the lowest ratings was “*Compared to when the trial started, the overall commitment required was similar to what I expected.”* On this item, seven participants (50% of those who completed the trial) reported that the overall commitment was somewhat more or much more than expected.

### Exploratory outcomes

**Fig 2:**
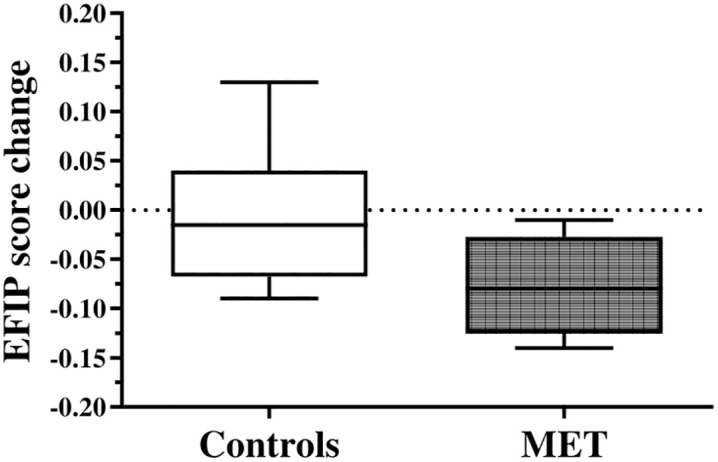
6-week changes in the primary exploratory outcome (frailty) as a function of group (boxplots). **Legend:** EFIP: evaluative frailty index for physical activity; MET: multimodal exercise training. **Note:** the dotted line (zero) represents no change in outcome from baseline to 6 weeks.

**Fig 3:**
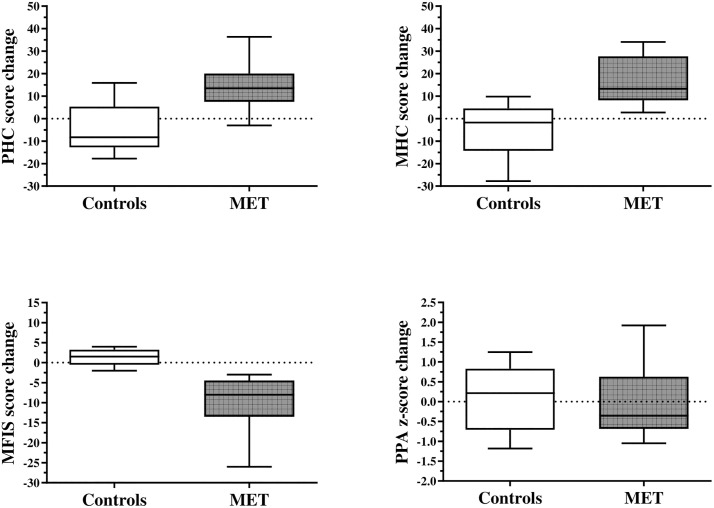
6-week changes in the secondary exploratory outcomes (quality of life, fatigue, and physiological fall-risk) as a function of group (boxplots). **Legend:** MET: multimodal exercise training; MFIS: modified fatigue impact scale; MHC: mental health composite score of the MSQoL-54 questionnaire; PHC: physical health composite score of the MSQoL-54 questionnaire; PPA: physiological profile assessment. **Note:** the dotted line (zero) represents no change in outcome from baseline to 6 weeks.

The within-group changes in exploratory outcomes, as well as the between-group differences in baseline to 6-week change, are fully summarized in Table 2. Figs 2 and 3 display the boxplots of the baseline to 6-week changes in the primary and secondary exploratory outcomes, respectively, as a function of group.

**Table 2:** Exploratory outcomes. Results are summarized as mean (standard deviation).

Outcomes	MET (n = 8)			Control (n = 6)			Between-group differences in baseline to 6-week change		
Primary	Baseline	6-week	Change	Baseline	6-week	Change	Mean	95% CI	Cohen’s d
EFIP score	0.27 (0.06)	0.19 (0.06)	−0.08 (0.05)	0.35 (0.08)	0.34 (0.05)	−0.01 (0.07)	−0.07	−0.14, −0.00	−1.21
**Secondary**									
PHC score	38.26 (11.37)	52.90 (19.03)	14.64 (11.42)	42.44 (15.38)	37.82 (15.55)	−4.63 (11.84)	19.26	5.61, 32.91	1.66
MHC score	49.95 (17.61)	66.44 (16.73)	16.48 (10.92)	58.67 (26.99)	53.91 (23.88)	−4.76 (12.99)	21.24	7.32, 35.16	1.80
MFIS score	46.38 (6.57)	36.25 (10.82)	−10.13 (7.43)	49.33 (8.82)	50.67 (9.54)	1.33 (2.16)	−11.46	−18.34, −5.13	−1.96
PPA z-score	2.36 (1.43)	2.38 (1.45)	0.02 (0.97)	1.85 (0.73)	1.96 (1.37)	0.11 (0.88)	−0.09	−1.19, 1.01	−0.09

**Abbreviations:** CI: confidence interval; EFIP: evaluative frailty index for physical activity; MET: multimodal exercise training; MFIS: modified fatigue impact scale; MHC: mental health composite score of the MSQoL-54 questionnaire; PHC: physical health composite score of the MSQoL-54 questionnaire; PPA: physiological profile assessment.

## Discussion

This study aimed to examine the feasibility of a novel MET program to reduce frailty in frail pwMS. The intervention satisfied the main criteria for feasibility, as the retention rate and proportion of completed training sessions were 87.5% and 97.2%, respectively. Other feasibility aspects, such as the absence of adverse events and training-related pain, the time spent in training, and the positive feedback provided by participants on the SPFQ indicate that MET was safe, acceptable, and well-received by frail pwMS.

A closer examination of the feasibility data suggests that, overall, our site had adequate resources for the conduct of the study. For instance, the achieved study timeline was aligned with the proposed timeline, and we did not have missing data in the exploratory outcomes. However, the recruitment rate (0.33 enrolled participants/week) was relatively low for an RCT [50]. Only 8.9% of approached individuals ultimately consented to participate, highlighting challenges for recruitment in future trials. The relatively low consent rate likely reflects the combination of the substantial time commitment associated with a supervised, facility-based, thrice-weekly intervention and the stringent inclusion criteria. For instance, we only included individuals who were between 40 and 65 years of age, meeting objective diagnostic criteria for frailty, and able to walk without bilateral support—a hard-to-reach population. Including frail pwMS was one of the most innovative aspects of our study, as the MET program was purposefully designed to counteract frailty in the ambulatory segment of the MS population. The upper age limit (65 years) was selected based on the inclusion criteria from our previous trial and to maximize safety [11,34]. The findings from this study indicated that MET was highly safe and feasible in the included population. Therefore, as a future direction, broadening the inclusion criteria to pwMS over 65 years would be a logical step. Indeed, frailty is associated with higher age in MS, [5] and frail older adults living with MS are a group of people likely to benefit from MET. Other strategies to maximize recruitment in future trials may include increasing flexibility in scheduling training sessions and engaging clinicians more directly in the recruitment process. Moreover, findings from the current investigation raise the possibility that a modified version of our MET program may represent a viable strategy to minimize frailty in the non-ambulatory segment of the MS population. As shown by previous research, upwards of 90% of pwMS with limited ambulation ability (PDDS score of 7) are frail [51]. Therefore, reducing the burden of frailty in this group of people is probably an important yet overlooked therapeutic goal. Several adaptations to promote MET among non-ambulatory pwMS (and ambulatory individuals with substantial mobility impairments), such as utilizing a recumbent stationary bike and RT, are possible. Further research to develop MET protocols to reduce frailty in non-ambulatory pwMS is warranted. In addition, from an implementation perspective, the recruitment challenges observed in this pilot study underscore the importance of optimizing accessibility and scalability of MET interventions for frail pwMS. While the current protocol required in-person, supervised training using specialized equipment, future implementation efforts may benefit from adapting MET components to community-based or clinical rehabilitation settings, or from leveraging tele-rehabilitation and lower-cost technologies where appropriate. Understanding and addressing barriers related to transportation, fatigue, and perceived burden will be critical for successful translation of MET into routine clinical practice should its efficacy be confirmed in larger trials.

The exploratory outcomes were promising, as all participants who were randomized to MET exhibited a reduction in EFIP scores after the intervention (range: −0.02 to −0.14, mean = −0.08, SD = 0.05). Previous research has suggested that reductions in frailty index measures greater than 0.03 represent a clinically meaningful change in frailty levels in older adults [52,53]. Interestingly, the mean change in EFIP found in the current study was greater than these values. However, a direct comparison with previous studies is not possible, as different frailty index measures were used, and the thresholds for clinically meaningful change are provided for contextual interpretation only. Further research is needed to establish what represents a clinically meaningful change in EFIP for frail pwMS. Importantly, participants randomized to MET also showed improvements in quality of life (both PHC and MHC of the MSQoL-54) and fatigue (total MFIS score), which are often seen as corollary measures of frailty. The mean pre-post changes in MSQoL-54 and MFIS exhibited by the experimental group were also consistent with a clinically meaningful change [54,55]. This provides evidence that our MET program satisfied the principle of pleiotropic benefit, showing potential to improve multiple health aspects impacted by MS. We should also note that the training duration for this study was relatively short, and it is quite possible that prolonging MET beyond six weeks may have resulted in a greater impact on the measured outcomes. Relatedly, no meaningful between-group changes in physiological fall-risk (PPA z-score) were observed over the 6-week intervention. This may reflect the short duration of training, suggesting that longer or higher-intensity MET programs may be needed to impact fall-risk in frail pwMS. Finally, the study findings also provide preliminary information that may inform the planning of a future trial. Although formal power calculations were not a primary objective of this feasibility study, the observed between-group differences and corresponding standardized effect size estimates for key outcomes such as EFIP, MSQoL-54, and MFIS may serve as an initial reference for sample size considerations. However, these estimates should be interpreted cautiously due to the small sample size and ideally supplemented with pragmatic considerations related to recruitment and retention when designing the next trial.

## Study limitations

The current investigation is not without limitations. First, the study findings should be interpreted with caution due to the small sample size, which may have affected the estimation accuracy of the 95% confidence intervals for the pre-post changes in outcomes. Second, the relatively small sample size raises the possibility that our inclusion and exclusion criteria may have been too stringent, as discussed previously. Relatedly, owing to the selected and hard-to-reach nature of the study population (particularly with respect to age range, ambulatory status, and objective frailty criteria), the generalizability of the findings to broader MS populations may be limited. Further refinements of the inclusion criteria may be needed prior to the next trial. In addition, we used a passive waitlist control group. This methodological approach was chosen to generate proof of concept feasibility data in this early phase of research. However, the lack of an active control group should be acknowledged as a limitation in terms of our ability to generate more robust preliminary efficacy data. Future trials will incorporate an active control group to allow for more robust evaluation of intervention-specific effects. We should also acknowledge that staff time and intervention-related costs were not formally evaluated in this study. Future trials should incorporate detailed assessments of personnel requirements and costs to better inform scalability and implementation of MET interventions. In addition, while feasibility thresholds for retention and adherence were pre-specified (>80%), formal go/no-go progression criteria were not defined for other feasibility outcomes, such as recruitment, safety, or user engagement. This should also be considered a study limitation. Moreover, deviations from the original analytical plan, including reclassification of pre-specified primary and secondary outcomes as exploratory and the use of descriptive rather than inferential statistics, were required by recruitment challenges and should be considered when interpreting the findings.

## Conclusions

This was the first study to examine the feasibility of MET to reduce frailty in frail pwMS. The findings indicated that MET was safe, feasible, and well-received by the study participants. In addition, our site demonstrated adequate resources for the conduct of the study, and refinements to the inclusion criteria to maximize recruitment for the next trial were identified. The promising results support the viability of MET for future larger-scale trials targeting frailty reduction in this population.

## Supporting information

S1 minimal dataset Minimal dataset. (XLSX)

S2 study protocol Study protocol. (PDF)

S3 IRB approval IRB approval. (PDF)

S4 CONSORT Checklist CONSORT checklist. (PDF)
