# Supplementary material for: Reducing frailty in frail people with multiple sclerosis: Feasibility of a 6-week multimodal exercise training program
Source: PLoS One. 2026 Apr 15;21(4):e0347063. doi: 10.1371/journal.pone.0347063 (PMC13082602; doi:10.1371/journal.pone.0347063)
Supplement: S2 study protocol — (PDF) [file pone.0347063.s002.pdf]

University of Kansas Medical Center
RESEARCH PROTOCOL INVOLVING HUMAN SUBJECTS
TEMPLATE WITH GUIDANCE

Version date: 01/30/2023

Principal Investigator: Tobia Zanotto, PhD

Study Title: Multimodal Exercise Training to Reduce Frailty in People with Multiple Sclerosis

Co- Investigator(s): Jacob Sosnoff, PhD; Hannes Devos, PhD; Sharon Lynch, MD

I. Purpose, Background and Rationale

A. Aim and Hypotheses

1. Frailty is a biological syndrome of decreased reserve and resistance to stressors arising from cumulative degradation across multiple physiologic systems.[1] Frailty is very common in individuals living with multiple sclerosis (MS), regardless of age and level of disability. People with MS (pwMS) have up to a 15-fold higher risk of being frail compared to age-matched individuals living without MS.[2-4] Frailty within MS is strongly associated with adverse clinical outcomes such as falls [5] and negatively impacts the quality of life. Consequently, there is a critical need *to* identify and evaluate strategies for counteracting frailty in pwMS. People with MS become frail at a younger age compared to individuals without MS.[4] This early onset of frailty may be an optimal window of opportunity for implementing exercise interventions aiming to reduce frailty.[6,7] Reducing frailty would have a significant impact on improving the health of pwMS. To date, however, it is not known whether exercise interventions can reduce frailty in MS. The purpose of this project will be to explore the feasibility and preliminary efficacy of a multimodal exercise training program to reduce frailty in pwMS.
2. The proposed project has two research aims and one exploratory aim:

   Aim 1: To determine the feasibility of a multimodal exercise training in pwMS.

   We hypothesize that at least 80% of participants will complete the multimodal exercise training and that no serious adverse events will be recorded during the training sessions. In addition to feasibility, we will also generate the effect sizes from the intervention, which will inform the development of a fully powered randomized controlled trial (RCT).

   Aim 2: To explore the effects of the multimodal exercise training on frailty in pwMS.

   We hypothesize that participants in the multimodal exercise group will have greater reduction in frailty index[19] than participants in the control group. Moreover, we expect that participants randomized to multimodal exercise will also exhibit a clinically meaningful change in the frailty index (i.e., >0.03)[21].

   Exploratory aim: To compare pain levels in pwMS with and without frailty, as well as to explore changes in pain before and after the exercise training.

We hypothesize that frail pwMS will have higher pain levels compared to the non-frail, and that participants randomized to the multimodal exercise training program will have a greater reduction in pain compared to participants randomized to the control group.

B. Background and Significance

1. Study Significance: This proposed investigation is designed to examine the feasibility of an exercise-based intervention to reduce frailty in people living with MS. The proposed research will be the first to establish the feasibility of targeting frailty in pwMS and will begin to examine the dynamic nature of frailty in MS. This project will also yield the effect sizes necessary to power a large scale RCT to reduce frailty in pwMS. These advances will pave the way for planned prospective studies toward effective targets for diagnostics, and rehabilitation of frailty in MS.

2. MS is a chronic, immune-mediated neurodegenerative disease that affects ~1 million individuals in the United States.[22] It is characterized by demyelination and neuronal loss.[23] MS is one of the most common causes of non-traumatic disability among adults of 20 – 40 years globally [24] and is second only to heart failure in direct all-cause medical costs for chronic conditions.[25] Early in the disease process there are acute episodes of neurological deficits (i.e., relapses) resulting from acute inflammatory demyelination. The location of the demyelinating lesions determines physiological system(s) affected and symptom presentation. MS is characterized by a progressive accumulation of disability, which typically manifests through walking impairment,[26,27] muscle weakness,[28] fatigue,[29] psychological problems[30], and cognitive impairment[31]. There is a critical need to identify new approaches to promote quality of life in the MS community.

   Frailty is a serious public health challenge that is poised to overwhelm global health care systems.[37] Frailty is a biological syndrome of decreased reserve and resistance to stressors arising from cumulative declines across multiple physiologic systems.[1] It is associated with reduced quality of life, falls, health care utilizations and early mortality.[38] The elevated risk of adverse outcomes can occur regardless of the presence of comorbidities in frail individuals.[39] Frailty is frequently characterized by reductions in muscle strength, reduced physical activity, fatigue, reduced gait speed and unintentional weight loss[18] (factors which are common in MS). Frailty is more than simply the accumulation of comorbidities and represents a dynamic condition that can range from non-frail (“fit”) to severely frail.[40] Building upon the dynamic nature of frailty, there has been a call to systematically investigate intervention strategies to reduce frailty[40].

   Although it is traditionally seen as an age-related phenomenon, frailty can occur throughout the lifespan, especially in individuals with chronic conditions, such as MS.[41] Given that MS impacts multiple physiological systems and is characterized by muscle weakness, gait impairment, fatigue and inactivity, it is not surprising that pwMS are frequently frail[2]. They have a 15-fold higher risk of being frail compared to age-matched individuals without MS.[4] Up to two thirds of ambulatory pwMS meet objective diagnostic criteria for frailty,[5] and this proportion is higher in individuals with more advanced disability[42]. The early onset of frailty in pwMS suggests that the etiology of this syndrome in MS differs from age-related frailty. Particularly, several key pathophysiological aspects of MS, such as chronic inflammation and neurodegeneration,[44] are likely to shape the

manifestation of frailty in people with MS.[45,46] Due to the unique features of frailty in MS, it is unclear whether frailty can be reduced in MS.

3. Literature Review: Several strategies to counteract frailty have been studied in older adults.[15,47-49] Most clinical trials have been conducted in community-dwelling older adults, wherein exercise interventions represented the main strategy, and physical function (e.g. gait and strength) measures were the main outcomes.[40] Overall, the investigations have reported reduced markers of frailty following exercise-based interventions.[50] Particularly, multicomponent exercise programs involving resistance training, gait and balance training represent the best strategy to improve hallmarks of frailty in frail individuals.[51]

   There is considerable evidence that physiological function in pwMS can be improved with targeted exercise interventions.[8,52] Progressive resistance training has a significant effect on muscle strength and walking performance in pwMS.[28,53] Exercise has also been shown to have a beneficial effect on fatigue in persons with MS.[11] The general benefit of exercise in pwMS, highlights the possibility that a well-designed program will reduce frailty in this population. Recently we completed an 8-week RCT focusing on a multicomponent exercise program involving virtual reality treadmill training (i.e., simultaneous training of gait, balance, and cognition) in over 100 pwMS and found an improvement in multiple frailty-related measures, such as gait speed, cognition, and depression following the intervention.[13,14] Nevertheless, in a preliminary secondary analysis, this intervention did not result in a clinically meaningful, nor statistically significant reduction of frailty in a small subset (n=20) of the studied sample ($F=1.48$, $p=0.244$, $\eta_p^2=0.095$). The limited effect on frailty is not surprising given that the intervention was not specifically designed to target frailty, nor was it an inclusion criterion. However, the promise shown by our program in enhancing several key moderators of frailty, seems to open the possibility that adding elements targeting frailty such as an evidence-based resistance training component will have a greater effect on reducing frailty in pwMS.

   Many approaches and scales to evaluate frailty exist.[54] The two main conceptualizations of frailty are *physical frailty*, as operationalized through the Fried phenotype,[18] and the deficit accumulation model (frailty index approach).[19] The Fried phenotype is the most commonly utilized measure of frailty and defines five core components of frailty: slowness, weakness, inactivity, exhaustion, and shrinkage. Individuals who meet at least three of these components are defined as frail. The strengths of this operationalization of frailty are its cost- and time-effectiveness, as well as its large evidence base in terms of predicting negative health outcomes in various clinical populations.[20] On the other hand, the *deficit accumulation model quantifies frailty on a continuum*, based on the presence or absence of health-related deficits. From an operational standpoint, this model utilizes a relatively high number of health-related deficit items (at least 30 are recommended) as the basis to calculate a frailty index.[55] Importantly, one of the main advantages of the frailty index is that its operationalization is easily adaptable to many pre-existing health datasets, which is a desirable feature in terms of clinical implementation. In addition, the health-related deficits used to calculate the index encompass various domains of function (e.g., physical, cognitive, psychosocial function, etc.), making the frailty index *a more comprehensive conceptualization of frailty*.[20] Another strength of the frailty index

approach is that it may be more suitable than the Fried phenotype to capture the dynamic nature of frailty, namely its modifiability in response to interventions.[56] Conversely, one of the challenges of the frailty index is that it often requires effort to identify a suitable number of health-related deficits without violating its guiding principles (described in Approach).[57] Owing to the several physiological systems affected by MS,[44,46,58] we recently postulated that using a comprehensive view of frailty, such as the frailty index approach, may be required to capture the complexity of frailty in pwMS, as well as to evaluate the fine-grained changes in frailty over time and in response to interventions.[5,57] However, more research is required to evaluate the psychometric properties (e.g., test-retest reliability) of the frailty index in pwMS.

C. Rationale

1. There is considerable evidence that physiological function in pwMS can be improved with targeted exercise interventions.[8,52] The general benefit of exercise in pwMS, highlights the possibility that a well-designed program will reduce frailty in this population. Our recently completed RCT consisting of a motor-cognitive rehabilitation intervention was successful in reducing hallmarks of frailty in pwMS. The promise shown by our program in enhancing several key moderators of frailty, seems to open the possibility that adding elements targeting frailty such as an evidence-based resistance training component will have a greater effect on reducing frailty in pwMS.
2. The main limitation of the current body of knowledge is the lack of evidence regarding the dynamic nature (i.e., modifiability) of frailty in MS. Additionally, it is not clear whether well-designed exercise-based interventions can reduce frailty in pwMS, and whether measures of frailty, such as the frailty index, are reliable (and therefore appropriate to evaluate the effects of interventions) in pwMS. Moreover, interventions in older adults designed to target frailty have had numerous methodological limitations, namely limited assessment of frailty prior to and post-intervention and lack of control groups.[40] The current project is designed to overcome these limitations and set the foundation for interventions to reduce frailty in MS.
3. This project aims to improve the treatment of MS. This project will help determine if well-designed exercise-based interventions can reduce frailty in pwMS, which could in turn improve the overall quality of life in pwMS.

II. Research Plan and Design

A. Study Objectives: The broad research goal of this study is to provide novel data regarding the potential efficacy of multimodal exercise to reduce frailty in pwMS and will set the foundation for our long-term goal of identifying novel interventions to improve the quality of life of persons with MS. The specific aims of the project will be to determine the feasibility of a multimodal exercise training in pwMS (Aim 1), and to explore the effects of the multimodal exercise training on frailty in pwMS (Aim 2).

B. Study Type and Design: Using a pilot single-blind randomized controlled trial (RCT) study design, ambulatory individuals living with MS will be allocated to 6 weeks of multimodal exercise training (n=30) or to a waitlist control group (n=30) (Figure 1).

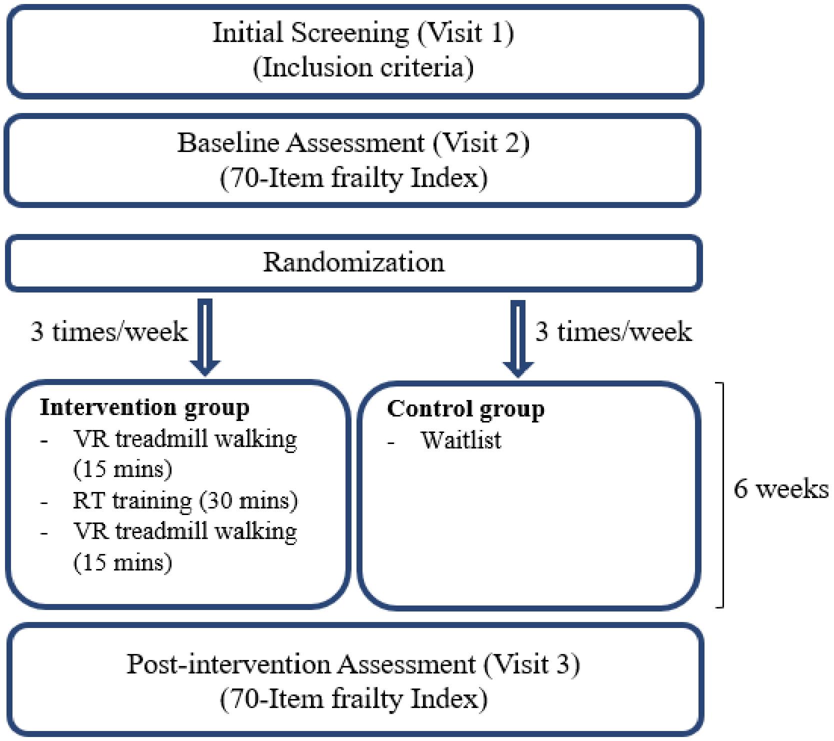

Figure 1. Study design.

The following outcomes will be collected to accomplish the study aims:

Aim 1: Feasibility Outcomes: The following outcomes, based on the guiding principles for feasibility studies,[83] will be utilized to examine the feasibility of the Virtual Reality and Resistance Training for MS (VRRT4MS) program in the study population: a) participant recruitment, i.e., number of consented individuals/number of approached individuals (expressed as a percentage); b) retention rate, i.e., number of participants completing the intervention/number of participants enrolled at baseline (expressed as percentage); c) appropriateness of data collection and training procedures, as quantified through the percentage of missing data for analysis purposes (i.e., missing items in the frailty index) and through time spent in training, respectively; d) participant safety, i.e., number of adverse and serious adverse outcomes throughout the study and self-reported pain, assessed by the numeric pain rating scale,[84] during each training session (the Borg[85] and Omni[86] scales of perceived exertion will also be administered throughout each training session to differentiate exertion from pain); e) evaluation of resources, i.e., difference between proposed and actual research timeline; f) user engagement, i.e., study participant feedback questionnaire. We note that the effects of VRRT4MS on the primary frailty outcome, namely the frailty index (AIM 2), will also represent a further feasibility outcome of the intervention.

Aim 2: Primary Frailty Outcomes: The primary frailty outcome of this investigation will be the frailty index.[19] This parameter was selected based on its significant potential to quantify fine-grained changes in frailty, compared to the Fried phenotype, and on our preliminary data on frailty in MS. Notably, a recent study in hospitalized older adults has identified a change in frailty index score > 0.03 as a minimum clinically important difference (MCID) in frailty status.[21]

Other measures: In addition to the frailty outcomes, we will also collect sociodemographic (i.e., age, gender, body mass index, marital and occupational status) and relevant clinical measures (i.e., number of prescribed medications, disease duration, MS type) at baseline.

C. Sample size, statistical methods, and power calculation

1. Descriptive statistics (frequency/percent and mean/SD) will be mainly used to summarize participants' characteristics and intervention outcomes. The means and 95% CIs for pre-post changes in the primary (i.e., frailty index) outcome will be estimated. No imputation will be considered for missing data. The Wilcoxon rank sum test will be used for comparing the pre-post changes in frailty index between the two groups. The intervention will be considered feasible if the retention rate and the proportion of completed sessions are more than 80%. Pre-generated randomization codes will be prepared by the biostatistician (Dr. He). The randomization ratio will be 1:1.
2. Dr. Abbas Tabatabaei, who will be the assessor for the study assessments, will be blinded to which group the participants have been allocated to. Participants will be aware of which group they are in. The study coordinator and Dr. Sosnoff will have access to the code to the blind. The code will not need to be broken unless there are unforeseen circumstances in which Dr. Abbas Tabatabaei needs to know which group a particular participant was assigned to. Following the baseline assessment (visit 2), participants will be randomized to one of the two groups based on pre-generated randomization codes prepared by the biostatistician, Dr. He. In accordance with the CONSORT guidelines,[59,60] sequence generation, allocation concealment and group allocation will be blinded and separate processes.
3. For this pilot study, we plan to enroll a convenience sample of 60 ambulatory people (30 per group) with Multiple Sclerosis living with frailty that meet inclusion/exclusion criteria. These subjects will provide feasibility data and preliminary efficacy data for designing a future study if the findings are promising. We expect a 15% dropout rate, and at that at least 24 subjects per group will complete the study.

D. Subject Criteria (See Vulnerable Populations appendix, if applicable): We will enroll 60 ambulatory pwMS living with frailty. During Visit 1, we will screen subjects for frailty based on established cut-offs according to the Fried phenotype.[18] This screening tool was chosen to ensure a time- and cost-effective identification of pwMS who are frail.[20]

1. Inclusion criteria: Subjects must be aged 40-65 years old to be enrolled in this study. Subjects must meet 3 of 5 Fried's criteria (weight loss; fatigue; weakness; ambulation; inactivity) to be considered. Subjects must have a confirmed diagnosis of MS by the treating neurologist, be free from MS relapses in the last 30 days, have an Expanded Disability Status Scale (EDSS) [61,62] score ≤ 6.0, and be fluent in spoken and written English.
2. Exclusion criteria: Subjects will be excluded from participation is they are unable to walk unassisted for at least ten meters, are diagnosed with other clinically important neurological conditions (such as Parkinson's Disease, epilepsy, etc.), exhibit severe cognitive impairment (as determined by a Mini-Cog score <3), and are diagnosed with active psychiatric problems. Additional exclusionary factors include unstable cardiovascular conditions (e.g., malignant arrhythmias, critical mitral stenosis, clinically severe left ventricular outflow obstruction, critical proximal coronary artery stenosis), arthritis of the lower limbs, acute lower back or lower limbs pain, rheumatic and/or severe orthopedic problems that may interfere with resistance training. Co-I Dr. Lynch

will confer with the research team to ensure that the inclusion/exclusion criteria are adequately implemented.

3. Withdrawal/Termination criteria: Participants could be withdrawn from the study due to a variety of circumstances. Participants may be withdrawn if they develop a condition deemed exclusionary. Participants may be withdrawn from the study if the PI, and/or the study team deem that it is in the participant's best interest to be withdrawn, or if a significant safety concern has been identified. If there are any concerns that the participant and/or study team will be at risk if the participant continues, they will be withdrawn. Some participants initiate withdrawal based on physical health or availability, or other personal choices.
4. Subjects currently enrolled in other exercise trials will not be considered eligible to participate.

E. Specific methods and techniques used throughout the study

1. Laboratory tests: Participants will be asked to complete a blood draw, in fasting conditions (in the morning), during study visits 2 and 3. The blood draws will be performed by a trained nurse at the Landon Center on Aging. The blood drawn will be done to assess biomarkers that are correlates of frailty such as NfL, BDNF, GFAP, CRP, IL-6, IL-10, TNF-alpha, IFN-gamma, and CXCL9, mi-RNAs. The amount of blood drawn will be approximately 12-15 mL.
2. Study Procedures: Participant involvement in this study will begin with the screening visit, where subjects will be screened for eligibility using objective diagnostic criteria. The screening visit will take place at the KU Center for MS Care and will take about 10-15 minutes. At the screening visit, participants will go through the informed consent with a study team member. If the participants consent, they will then complete the Mini-Cog questionnaire and the Fried phenotype to determine if they meet the frailty criteria. The Fried phenotype evaluates 5 components: fatigue, weakness, ambulation, inactivity, and loss of weight. Fried scores range from 0-5 (i.e., 1 point for each component). Higher scores reflect higher frailty. Subjects who total at least 3 points out of 5 will be classified as frail and will be eligible to participate. The Fried phenotype will also be repeated after the intervention, during Visit 3.

   Once participants meet the screening criteria, they will attend the first study assessment (Visit 2 – baseline assessment). During this visit, participants will undergo the blood draws in the morning, in fasting conditions (i.e., at least 12 hours of fasting), at the Landon Center on Aging (laboratory tests listed in Table 1 – see biomarkers). After the blood draw, participants will complete a comprehensive battery of physical [18,65-70,94] and cognitive tests,[71,72] self-reports,[73,74,93] clinical measurements,[75,76] and nutritional status measures[78-80] (see Table 1) in the Human Performance Laboratory at the Landon Center on Aging. These procedures will take about 90 minutes. At the end of the visit, they will be provided with a wearable sensor consistent with our previous work.[81] From these assessments, we will derive 70 health-deficit items that will be used to calculate a frailty index according to the guiding principles of the deficit accumulation model.[19,82] A synopsis of the frailty index is provided in Table 1. The health-deficit items will be coded on a 0-1 scale, to indicate the absence or presence of health deficits, using standard validated procedures[55] by a single researcher experienced in frailty assessments and blinded to group allocation (PI Zanotto). The frailty index score will be calculated as the

sum of all deficit-related scores divided by the sum of the total number of possible health deficits. The same procedures will be repeated during the post-intervention assessment (Visit 3). During the screening visit and visit 3, participants will complete two pain questionnaires and a pain threshold test. First, participants will complete the Paindetect, a screening tool of neuropathic pain (one of the most common pain pwMS) [95] and the Fibromyalgia Survey Criteria (a proxy measure of nociplastic pain). Then, participants will undergo a pressure pain threshold involving the middle part of bulk of medial and lateral gastrocnemius muscles[96] as well as the thumbnail of the participant's dominant hand[96]. This is measured with a digital algometer, which has been shown to have good reliability in the gastrocnemius region[96]. The digital algometer is made up of a 1-cm-wide disk located on the middle part of bulk of medial and lateral head of gastrocnemius muscles. The algometer's pressure will be increased gradually (with a speed approximately 1 N/cm2 per second) and will be stopped immediately when the participant reports the transition from discomfort to pain. The average value of three measurements will be recorded.

Table 1. Synopsis of the 70-item frailty index: Domains, sources and measures utilized as health deficit items.

Domains of frailty	Items, number	Sources	Measures
Global health	2	MSQoL-54	self-reported health.
Function in ADLs	6	MSQoL-54	Questions about the ability to perform ADLs
Energy	5	MSQoL-54, MFIS	Questions about tiredness, energy, and fatigue
Psychosocial function	6	MSQoL-54, MFIS	Questions about participation in social activities, feelings of happiness, downheartedness, etc
Health attitudes/sleep quality	6	Wearable sensor (worn for three consecutive days)	Number of daily steps, number of 30-seconds walking bouts, moderate to vigorous physical activity, total physical activity, number of hours spent sleeping, sleep efficiency.
Cognition	6	CVLT-II, SDMT, BVMT, TMT, MFIS	Total words recalled, Total BVMT score, Raw SDMT score, TMT times, self-reported memory problems.
Physical performance	6	Instrumented gait analysis, 6MWT, Dynamometry	Self-selected gait speed, functional ambulation profile, 6MWT distance, handgrip strength, knee extensor strength, 5X chair stand.
Nutritional status	5	Laboratory tests, Self-report.	BMI, serum albumin, appetite disorders, problems with cooking.
Comorbidities	16	Medical records, Automated BP monitor, MSQoL-54.	Polypharmacy, systolic BP, diastolic BP, comorbidities; fall history
Biomarkers	12	Laboratory tests	NfL, BDNF, GFAP, CRP, IL-6, IL-10, TNF-alpha, IFN-gamma, and CXCL9, mi-RNA**s**.

Legend: ADL: activities of daily living, MSQoL-54: 54-item multiple sclerosis quality of life questionnaire, MFIS: modified fatigue impact scale, CVLT-II: California verbal

learning test, SDMT: symbol digit modalities test, BVMT: brief visuospatial memory test; TMT: trail making test, 6MWT: 6-minute walk test, BP: blood pressure, BMI: body mass index, CRP: c-reactive protein.

All study outcomes will be assessed in a standardized manner during Visit 2 and 3. Following visit 2, participants will be randomized to one of the two groups based on pre-generated randomization codes prepared by the biostatistician. In accordance with the CONSORT guidelines,[59,60] sequence generation, allocation concealment and group allocation will be blinded and separate processes. Participants will then either complete the waitlist period (passive control) or attend the exercise training visits 3 times a week, for 6 weeks, totaling 18 training visits. Each training visit will last one hour. The training visits will take place at the Laboratory for Advanced Rehabilitation Research in Simulation at the University of Kansas Medical Center.

Intervention (Multimodal exercise) Group: Participants will complete 15 minutes of virtual reality treadmill training (VRTT), followed by 30 minutes of progressive evidence-based resistance training (RT),[16,17] followed by other 15 minutes of VRTT. This interval training design was chosen to induce acute fatigue[88,89] before the last bout of VRTT. See Table 2.

Table 2. The Multimodal exercise program.

Exercise components	Duration	Description	Target (frailty domains)*
1) VR treadmill training	15 minutes	Treadmill walking with interactive virtual environment.	- Slowness<br>- Cognition
2) RT	30 minutes	Seated leg press, seated hamstring curls, seated calf raises, seated chest press, seated lat-pulldowns, seated shoulder press.	- Muscle weakness<br>- Shrinkage
3) VR treadmill training	15 minutes	Treadmill walking with interactive virtual environment.	- Slowness, Cognition<br>- Exhaustion

Legend: VR: virtual reality, RT: resistance training, * The multimodal exercise program will also inherently target the 'inactivity' component of frailty.

Both the VR and RT components of the program will be individualized based on the participant's abilities and the training progression will be adjusted every 2 weeks.[13,17] Over-ground gait speed will be assessed prior to the intervention and every two weeks thereafter and will be utilized to set the treadmill speed for the VR component. As part of VR, participants will navigate through a virtual environment projected on a TV screen while receiving feedback from the system (Figure 2. The RT component will consist of performing evidence-based RT exercises, as fully described in Table 2. In the event participants are unable to complete 15 minutes of continuous walking, walking duration will be progressively increased in 1–3-minute bouts until the prescribed time is reached. The estimated 1-repetition maximum (1-RM) of all RT exercises will be evaluated using validated standard procedures (for untrained individuals) [90] prior to the intervention. At the beginning of the intervention, participants will complete 3 sets of 8-12 repetitions at 20-30% of the 1RM and slowly progress up to 80% over the course of 8 weeks.[16] The end point of each set will always be limited to volitional fatigue. Recovery periods of 2-4 minutes will be prescribed between sets and exercises.

Control Group: Participants randomized to the control group will be put on a waitlist (passive control group) for 6 weeks. Upon completion of Visit 3, participants in the control group will be able to receive the intervention, if they wish so.

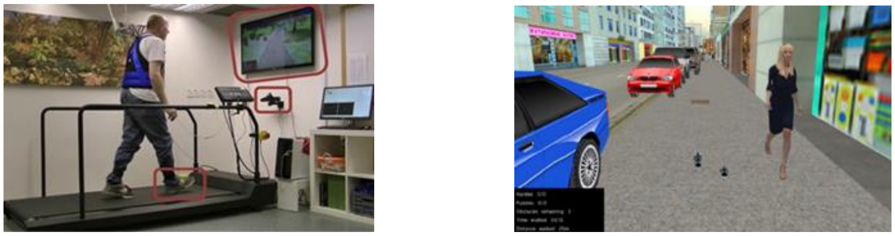

Panel A Panel B

Figure 2. Gait Better (TM) virtual reality (VR) treadmill training set up. Panel A. Camera detects markers on feet and projects them on screen. Panel B. View of VR monitor depicting individual's virtual feet, visual distractors, and obstacles.

3. All procedures, tests, and study visits will be performed solely for research purposes. Therefore, participants and their insurance companies will not be billed.
4. The blood samples taken in the study will be labeled with the participant's study ID as a way of maintaining confidentiality, so the blood sample will not be traceable to the participant's name. The blood sample will be destroyed by researchers after the participant's involvement in the study is complete and data have been analyzed. The blood samples will be stored in a secure area in Breidenthal.
5. Timeline: This project will be completed in 2 years. In months 0-2, we will obtain IRB approval, prepare case report forms and train staff. The study will begin in month 2. For a given participant the study will take 2.5 months. We will aim to recruit and train 7-8 participants in 4-month cycles starting in month 2. All participants will have completed training and the post-intervention assessment (month 18) after 4 cycles (4 cycles x 7-8 participants ~ 30 participants). This approach will yield a 6-month buffer to allow for unanticipated delays. Analyses of the unblinded data and preparation of manuscripts/funding applications will take place in months 18-24.

F. Risk/benefit assessment:

1. **Physical risk:** The main risk of this study would be risks typically associated with exercise. Participants may experience shortness of breath, muscle soreness, dizziness, and increased heart rate. These effects typically resolve shortly after the exercise is stopped, however, muscle soreness may last a few days. Given the prevalence of fatigue in persons with MS, it is possible that participants will become fatigued during study visits. Additionally, the blood draw may present a risk to participants. Participants may experience momentary discomfort and bruising. Infection, excess bleeding, clotting and fainting are also possible although their occurrence is unlikely. Participants may also be at risk of falling during the exercise training. Injuries sustained from falling could range from mild, such as scratches and bruises, to severe, such as head injuries and/or bone fracture. There is also the risk of a data breach or unintentional disclosure, which is a risk to a participant in ANY research study. There is a risk of contracting COVID-19, which is also present in any research which involves face-to-face interaction between humans. Finally, the pressure pain threshold test may be uncomfortable for some individuals.

   The first risk identified was the risk of incurring symptoms typically experienced during exercise. This risk will be mitigated in a variety of ways. Participants will be allowed breaks whenever they need it and be given access to water. Participants will only be

asked to complete exercises that they feel comfortable with. Additionally, the intervention program will be individualized based on the participant's abilities and will be readjusted every two weeks. Given the high rate of fatigue in persons with MS, it is possible that participants will become fatigued during study visits. To mitigate the risk of fatigue, participants will be given ample opportunity for rest during study visits. It is also likely that participants in the intervention group will experience temporary muscle soreness due to the resistance training aspect of the program. Each exercise session will begin and end with stretching to further minimize the risk of muscle pain. In addition, we will monitor perceived exertion through the Borg and Omni scales during each training session. We will also evaluate pain through the numeric pain rating scale during each training session (to differentiate this symptom from perceived exertion). If a participant reports a score higher than 3 on the numeric pain rating scale, we will stop the training session. To minimize the risk of musculoskeletal discomfort during maximal strength testing (1-RM assessment), we will use validated standard procedures for untrained individuals. To prevent participants from falling, participants will be encouraged to take a break if they feel tired, unwell, or dizzy. Participants will be spotted by a member of the research team and will wear an overhead harness that can be used to stabilize a participant if they were to become unsteady. In terms of the risks presented by the blood draw, the researchers will work to mitigate those risks by having the blood draw performed by trained clinicians in a sterile, cleaned environment, therefore minimizing risks.

Another risk identified was a data breach or unintentional disclosure. To prevent against this risk, several considerations have been made. Firstly, study personnel will receive HIPAA and information security training offered by the university. Any sensitive information will be stored in drives approved by the university for handling sensitive information. Participant information will also be stored in the Clinical Research Information System (CRIS), a system widely used by researchers, which offers secure protection for sensitive information. Any information that is not digital will be stored in secure locations by either the PI or study coordinator and will be secured by lock. Either the PI or study coordinator will hold the lock. Finally, the study team will only access protected information on a "as needed" basis. Protected information not relevant to the study will not be accessed by the study team. Participants will be informed of who will have their protected information and how it will be handled in the informed consent.

Another risk identified is the possibility of contracting or spreading COVID-19. This risk is present in any research where human beings meet face-to-face, even if the research does not involve human participants. The study team will follow all procedures, practices, and guidance set into place by the university. The study team will be fully vaccinated (including a booster shot). Masks will be worn according to university policy as well as researcher/participant comfort levels. Surfaces frequently touched will be cleaned and sanitized regularly. Hand sanitizer will be readily available in the lab and used by study team personnel and encouraged in participants any time there may be close contact between researchers and participants. 6-foot distancing will be followed when possible. Equipment used by participants (such as a safety harness) will be disinfected between uses.

The final risk that was identified is discomfort during the pressure pain threshold test. This will be mitigated by interrupting the test as soon as the participant reports any pain.

We also note that there were no serious adverse events in the 6-week virtual reality intervention in 108 persons with MS which serves as the foundation for the intervention

group. The safety of our approach is consistent with a systematic review highlighting the safety of exercise in persons with MS.

2. Psychological risk: None Known
3. Social risk: None Known
4. Economic risk: None Known
5. Potential benefit of participating in the study
   a. As an interventional study, this proposal may provide benefits to participants. If the intervention is successful, participants assigned to the intervention could have an improvement in their frailty, which could improve health outcomes. Furthermore, participants assigned to the control group may also experience benefits associated with increased exercise if they decide to participate in the intervention after their final assessment. Benefits for both groups could include reduced fatigue, increased muscle strength, improved walking speed, and improved endurance. Additional benefits to participants can include a sense of pride for contributing knowledge to the greater scientific community, assisting researchers in better understanding their disease, and giving researchers information key to improve health outcomes in people with MS. Finally, participants will get to feel a greater sense of belonging to the MS community.
   b. The proposed work will allow us to contribute more knowledge to the current literature regarding frailty and MS. The proposed work could allow us to contribute evidence regarding the dynamic nature of frailty in MS. It is not clear whether well-designed exercise-based interventions can reduce frailty in people with MS. Moreover, interventions in older adults designed to target frailty have had numerous methodological limitations – namely limited assessment of frailty prior to and post-intervention and lack of control group. The current study is designed to overcome these limitations and set the foundation for interventions to reduce frailty and maximize quality of life in pwMS.
   c. While this research primarily benefits the MS community, additional benefits could be seen in science's general understanding of aging. Investigating why people with MS become frail earlier than people in other populations may inform how we view and understand frailty in general. Additionally, our investigation into exercise training to reduce frailty may not just benefit pwMS, but other individuals suffering from frailty as well. For humanity in general, this research could provide further evidence of the positive benefits that exercise can have on the human body.

G. Location where study will be performed: The screening visit will be performed at the KU Center for MS Care. The assessments (visits 2 and 3) will be performed in the Landon Center on Aging. The training visits will be performed at the laboratory for Advanced Rehabilitation Research in Simulation at KUMC.

H. Collaboration (with another institution, if applicable): N/A

I. Single IRB Review for a Multi-site study (if applicable): N/A

J. Community-Based Participatory Research (if applicable): N/A

K. Personnel who will conduct the study, including:

1. Indicate, by title, who will be present during study procedure(s): PI, Co-I, study coordinator, research technician, and GRAs.
2. Primary responsibility for the following activities, for example:
    a. Determining eligibility: Dr. Lynch, study coordinator with assistance from PI (Dr. Zanotto).
    b. Obtaining informed consent: PI (Dr. Zanotto) and Dr. Abbas Tabatabaei
    c. Providing on-going information to the study sponsor and the IRB: PI (Dr. Zanotto), study coordinator
    d. Maintaining participant's research records: study team
    e. Completing physical examination: GRAs
    f. Taking vital signs, height, weight: GRAs
    g. Drawing / collecting laboratory specimens: Landon Center on Aging
    h. Performing / conducting tests, procedures, interventions, questionnaires: Dr. Tabatabaei and GRAs.
    i. Completing study data forms: study team
    j. Managing study database: study coordinator and PI (Dr. Zanotto)

L. Assessment of Subject Safety and Development of a Data and Safety Monitoring Plan

a. Given that the proposed investigation utilizes a randomized controlled trial study design, we will have an independent Data and Safety Monitoring Board (DSMB) for the trial. The primary responsibility of the board will be to protect the safety of the research participants and ensure adherence to human subject protection policies. Secondary responsibilities will be related to the quality and validity of research protocols and data quality. The DSMB will be identified prior to the start of the research project. The members of the DSMB will likely include experts in multiple sclerosis research, exercise, medicine, biostatistics, and ethics. The Data and Safety Monitoring Board (DSMB) will include at least three people who are independent of the trial and have one or more of the following qualifications: physicians or researchers with experience in clinical trials related to frailty or exercise in people with multiple sclerosis. The DSMB members will elect a Chairperson and a Safety Officer (SO). The Chairperson and the SO may be the same person. Once the members of the DSMB have been identified, the Institutional Review Board will be notified of the membership of the DSMB and its specific responsibilities. The report of the DSMB will follow the template of the National Institutes of Health: protocol synopsis, study timetable, action items from prior DSMB meetings, protocol changes, enrollment (actual versus expected), screen failures, protocol deviations, demography, baseline characteristics, treatment duration, adverse events, and serious adverse events. We propose that the DSMB have one in-person meeting and one mid-year teleconference during years 1-2. Other meetings will be convened at the request

of the committee. The members of the DSMB will also receive regular data reports at a schedule agreed upon by the members. Types of analyses to be performed: Analysis of completion, accuracy, and correct and timely response to events, data quality, protocol-adherence and attrition. Safety-related triggers that would cause the PI to stop or alter the study: unanticipated serious adverse outcome to participant. The major tasks of the DSMB will be to (1) assess whether there are safety findings that require stopping or modifying the study and (2) adjudicate Serious Adverse Events and deaths that are unexpected and/or atypical for healthy young-old adults. The DSMB will receive un-blinded safety data quarterly. If the DSMB detects safety concerns, then the DSMB may request safety data more frequently. If, at any time during the study, the perceived risk/benefit of treatment changes, the DSMB has the authority to recommend protocol modification or study curtailment. During the course of the trial, the DSMB will receive information from concomitant trials performed or published elsewhere. In addition, the DSMB will receive periodic reports about recruitment and safety information. Based on information received during the trial, the DSMB may choose to recommend changes to the protocol. If protocol changes are necessary, the implementation of changes will avoid unmasking the accumulated outcomes to the clinical trial personnel who are responsible for the ongoing performance of the trial. If there are unforeseen safety hazards that emerge during the clinical trial, then the Data and Safety Monitoring Board will have the authority to recommend temporary or permanent cessation of the trial.

   b. Furthermore, the Principal Investigator (PI) will be responsible for ensuring participants' safety on a daily basis. Trained research personnel will perform monitoring weekly to assess for data entry errors, outliers, and missing data. Personnel will also perform audits quarterly to assess adherence to the protocol, informed consent documents, source documents, regulatory documents, signature lists, site delegation authority lists, screening lists and enrollment logs. The appointed study monitor will sign the monitor log and submit a monitor report to the PI. The research personnel will use multiple techniques to minimize missing data, losses to follow up, and non-response bias. In summary, the investigators will ask participants to provide primary and back-up contact information. In addition, the protocol permits assessment of outcomes telephone survey, and postal survey if the participant declines a face-to-face visit. The investigators will create Standard Operating Procedures for electronic data entry, data access, data back-up, and data security.

2. The investigators will use the definitions for "Adverse Event" and "Serious Adverse Event" as defined by the International Council for Harmonization. Data from the study will be monitored on a continuous basis by the PI, and co-investigators. All serious adverse events (SAEs), adverse events (AEs), will be reviewed by the PI on an ongoing basis. Participants will be encouraged to report any discomfort or adverse outcomes they experience during each study visit. Trained research assistants will record any AE/SAE during the intervention. The study team will report SAEs and AEs to the PI as soon as possible.
3. Research personnel will ascertain adverse events from the participant by non-directive questions during the research visits. In addition, participants may voluntarily report

adverse events. Research personnel will record adverse events on the case report form. The investigators will score the adverse event in terms of:

Severity: mild, moderate, or severe.
Clinically significant: yes or no.
Requires therapy: yes or no.
Relationship to the experimental interventions: suspected or not suspected.
Duration of the adverse event: start date, end date, or ongoing at visit.
Serious adverse event: yes or no.

When research personnel learn that a participant has experienced an adverse event, research personnel will contact the PI on the same day to receive instructions for the transfer of the participant for treatment and follow-up of the adverse event. In addition, research personnel will complete an adverse event form. Participants with adverse events are eligible to remain in the trial unless there is withdrawal of consent. Participants who have experienced a serious adverse event will be withdrawn from the study.

The investigator will communicate with the participant and primary care physician (if deemed necessary by PI) to acquire information to complete the treatment questions on the adverse event form. The specific questions are: no action taken (i.e., further observation only); intervention temporarily interrupted; intervention permanently discontinued due to this adverse event; patient hospitalized. The investigator will continue to communicate with the participant and primary care physician until resolution to record change of severity, change of suspected attribution to intervention, and change of treatments for the adverse event.

The investigators and the Data and Safety Monitoring Board will review Adverse Events and Serious Adverse Events. If unexpected experimental-related adverse events emerge during the course of the trial, then the investigator will report the adverse events to the Institutional Review Board and the National Institutes of Health within the timelines required by each entity.

III. Subject Participation

A. Recruitment:

1. Participants will be recruited from the KU Center for MS Care.
2. Dr. Lynch, as the Medical Director of the KU Center for MS Care will recruit through her clinics and patients. Other members of the study team, particularly the PI and research technician, will also be involved in recruitment.

B. Screening Interview/questionnaire: The screening visit will be conducted at the KU Center for MS Care by Dr. Lynch, the PI and a GRA. Dr. Lynch will pre-screen potential participants for medical history and EDSS inclusion/exclusion criteria before subjects are approached for recruitment. The PI and Dr. Tabatabaei will administer the Mini-Cog questionnaire and the Fried phenotype to complete the screening.

C. Informed consent process and timing of obtaining of consent

1 The PI (Dr. Zanotto) will give participants a detailed and comprehensive information about the study and will obtain their written consent, with assistance from Dr. Tabatabaei.
2 The consenting process will take place in a private room in the Landon Center on Aging, to give the participant privacy. The participant will be sent the informed consent form via email prior to the assessment visit so they have time to review the document prior to consenting, if they wish. The participant will be encouraged to ask as many questions as needed and will be reminded that they have as much time as needed to make a decision. The participant will be given a copy of the signed informed consent. Original copies will be stored securely in a locked cabinet and virtual copies of the document will be stored in a secure location such as a P: drive or REDCap.
3 We expect all of our participants to be able make the informed consent decision themselves. Dr. Lynch will use her clinical expertise to determine if patients she recruits are able to make their own decisions. Additionally, subjects with severe cognitive impairment (as determined by a Mini-Cog score <3) will be excluded from the study to ensure that participants are able to consent for themselves. If the PI, during the informed consent process, feels that the subject is unable to consent for themselves, they will be excluded from the study.

D. **Alternatives to Participation:** Subjects will be reminded that participation in this research project is completely voluntary and they can decide to not participate and/or to withdraw consent at any point without giving a reason, and without their care being affected.

E. **Costs to Subjects:** There will be no costs to participants in this research project.

F. **How new information will be conveyed to the study subject and how it will be documented:** Subjects will be informed of new information through a call from the study team. The study team will document the call and the participant's response.

G. **Payment, including a prorated plan for payment:** Participants will receive up to $190 dollars if they participate in the entire study. Participants will receive $50 for each of the following visits: the baseline assessment (Visit 2) and the post-training assessment (Visit 3). The participants will receive $5 for each of the 18 training visits. Participants will only be paid for the visits they have completed. If a participant drops out or withdraws, they will only be paid for the visits they have already attended. Participants will be paid via Clincard.

H. **Payment for a research-related injury:** Participants will not be able to pursue financial compensation should they be injured as a result of participating in this research. However, they are not precluded from seeking to collect compensation for injury related to malpractice, fault, or blame on the part of those involved in the research, including the institution.

IV. Data Collection and Protection

A. Data Management and Security:

1. The study team will have access to the study data. Access to data will vary according to study team member role. The PI will have the highest-level access to data.

2. Subject confidentiality will be maintained by assigning participants a study ID so that records will not be directly linkable to them (see below). Records will be retained per university policies.
3. Human subjects will be identifiable through coded information. Subjects will be assigned a screening and study ID.
4. Only the PI and Co-I Sosnoff will have access to the key to the code, or the "master key."
5. Participants will be given a unique study ID to protect their confidentiality. Except for the master key, no other data will have a direct link between the participant's name and the study ID. Therefore, participant names will not be on data, but rather, their study ID. Participants will have a screening ID, and once they pass the screening, a study ID.
6. High-risk data will be stored on either a P: drive provided by the university or on REDCap. Medium risk data will be stored on a university S: drive and on REDCap. Paper copies of study forms will be stored in locked cabinets.
7. Mobile devices will not be used for data collection or storage.
8. Identifiable data will not be sent outside KUMC.

B. Sample / Specimen Collection: Blood samples will be collected by trained nurses at the Landon Center on Aging (LCOA) following standard procedures. The blood samples will be destroyed immediately after the analysis has been conducted. LCOA staff will share the results of the blood analysis only with the research team and will use the study ID rather than the participants' personal information.

C. Tissue Banking Considerations: N/A.

D. Procedures to protect subject confidentiality: Participant confidentiality will be maintained by performing informed consent and procedures within a private space at the LCOA. Participants will be assigned a unique study identification number, and all study forms will be de-identified. These forms will only contain the unique study identification number, thereby ensuring participant confidentiality. After the completion of the study, other researchers will have access to the de-identified study data. This will enable others to address additional questions and further investigate the results of the interventions.

E. Quality Assurance / Monitoring

1. Trained research personnel will perform monitoring weekly to assess for data entry errors, outliers, and missing data. Personnel will also perform audits quarterly to assess adherence to the protocol, informed consent documents, source documents, regulatory documents, signature lists, screening lists and enrollment logs. The option to mark data forms for missing or incorrect data will be utilized in REDCap.
2. Third party monitoring. N/A.

V. Data Analysis and Reporting

A. Statistical and Data Analysis: Descriptive statistics (frequency/percent and mean/SD) will be mainly used to summarize participants' characteristics and intervention outcomes. The means and 95% CIs for pre-post changes in the primary (i.e., frailty index) outcome will be estimated. No imputation will be considered for missing data. The Wilcoxon rank sum test will be used for comparing the pre-post changes in frailty index between the two groups.

B. Outcome: The intervention will be considered feasible if the retention rate and the proportion of completed sessions are more than 80%. We expect that at least 80% of participants will complete the multimodal exercise training and that no serious adverse events will be recorded during the training sessions. We expect that participants in the experimental group will have greater reduction in frailty index than participants in the control group. Finally, we expect that participants randomized to multimodal exercise will also exhibit a clinically meaningful change in the frailty index.

C. Study results to participants: A short, written letter of the study results will be sent to participants once the study has concluded.

D. Publication Plan: Findings from this study will be presented at scientific conferences and published in research journals.

VI. Bibliography / References / Literature Cited

1. Rockwood K, Howlett SE. Fifteen years of progress in understanding frailty and health in aging. *BMC Med*. 2018;16:220.
2. Ayrignac X, Larochelle C, Keezer M, Roger E, Poirier J, Lahav B, et al. Frailty in ageing persons with multiple sclerosis. *Mult Scler*. 2021;27:613-620.
3. Belvisi D, Canevelli M, Baione V, Buscarinu MC, Pellicciari G, Fantozzi R, et al. Operationalization of a frailty index in patients with multiple sclerosis: A cross-sectional investigation. *Mult Scler*. 2021;27:1939-1947.
4. Hanlon P, Nicholl BI, Jani BD, Lee D, McQueenie R, Mair FS. Frailty and pre-frailty in middle-aged and older adults and its association with multimorbidity and mortality: a prospective analysis of 493 737 UK Biobank participants. *Lancet Public Health*. 2018;3:e323-e332.
5. Zanotto T, Galperin I, Mirelman A, Yehezkiyahu S, Estes J, Chen L, et al. Frailty and Falls in People Living With Multiple Sclerosis. *Arch Phys Med Rehabil*. 2022;103:952-957.
6. Gill TM, Gahbauer EA, Allore HG, Han L. Transitions between frailty states among community-living older persons. *Arch Intern Med*. 2006;166:418-23.
7. Spiers GF, Kunonga TP, Hall A, Beyer F, Boulton E, Parker S, et al. Measuring frailty in younger populations: a rapid review of evidence. *BMJ Open*. 2021;11:e047051.
8. Amatya B, Khan F, Galea M. Rehabilitation for people with multiple sclerosis: an overview of Cochrane Reviews. *Cochrane Database Syst Rev*. 2019;1:CD012732.
9. Baird JF, Sandroff BM, Motl RW. Therapies for mobility disability in persons with multiple sclerosis. *Expert Rev Neurother*. 2018;18:493-502.
10. Sandroff BM. Exercise and cognition in multiple sclerosis: The importance of acute exercise for developing better interventions. *Neurosci Biobehav Rev*. 2015;59:173-83.
11. Heine M, van de Port I, Rietberg MB, van Wegen EE, Kwakkel G. Exercise therapy for fatigue in multiple sclerosis. *Cochrane Database Syst Rev*. 2015;9:CD009956.
12. Brenner P, Piehl F. Fatigue and depression in multiple sclerosis: pharmacological and non-pharmacological interventions. *Acta Neurol Scand*. 2016;134:47-54.
13. Hsieh KL, Mirelman A, Shema-Shiratzky S, Galperin I, Regev K, Shen S, et al. A multi-modal virtual reality treadmill intervention for enhancing mobility and cognitive function in people with multiple sclerosis: Protocol for a randomized controlled trial. *Contemp Clin Trials*. 2020;97:106122.
14. Galperin I, Mirelman A, Schmitz-Hübsch T, Hsieh KL, Regev K, Karni A, et al. Treadmill Training with Virtual Reality for Gait and Cognitive Function among People with Multiple Sclerosis: randomized controlled trial. *Journal of Neurology* (Under Review - 2022).

15. Cameron ID, Kurrle SE. Frailty and Rehabilitation. *Interdiscip Top Gerontol Geriatr*. 2015;41:137-150.
16. Coelho-Júnior HJ, Uchida MC, Picca A, Bernabei R, Landi F, Calvani R, et al. Evidence-based recommendations for resistance and power training to prevent frailty in community-dwellers. *Aging Clin Exp Res*. 2021;33:2069-2086.
17. Fragala MS, Cadore EL, Dorgo S, Izquierdo M, Kraemer WJ, Peterson MD, et al. Resistance Training for Older Adults: Position Statement From the National Strength and Conditioning Association. *J Strength Cond Res*. 2019;33:2019-2052.
18. Fried LP, Tangen CM, Walston J, Newman AB, Hirsch C, Gottdiener J, et al; Cardiovascular Health Study Collaborative Research Group. Frailty in older adults: evidence for a phenotype. *J Gerontol A Biol Sci Med Sci*. 2001;56:M146-56.
19. Rockwood K, Mitnitski A. Frailty in relation to the accumulation of deficits. *J Gerontol A Biol Sci Med Sci*. 2007;62:722-727.
20. Dent E, Kowal P, Hoogendijk EO. Frailty measurement in research and clinical practice: A review. *Eur J Intern Med*. 2016;31:3-10.
21. Theou O, van der Valk AM, Godin J, Andrew MK, McElhaney JE, McNeil SA, et al. Exploring Clinically Meaningful Changes for the Frailty Index in a Longitudinal Cohort of Hospitalized Older Patients. *J Gerontol A Biol Sci Med Sci*. 2020;75:1928-1934.
22. Wallin MT, Culpepper WJ, Campbell JD, Nelson LM, Langer-Gould A, Marrie RA, et al; US Multiple Sclerosis Prevalence Workgroup. The prevalence of MS in the United States: A population-based estimate using health claims data. *Neurology*. 2019;92:e1029-e1040.
23. Thompson AJ, Banwell BL, Barkhof F, Carroll WM, Coetzee T, Comi G, et al. Diagnosis of multiple sclerosis: 2017 revisions of the McDonald criteria. *Lancet Neurol*. 2018;17:162-173.
24. Dobson R, Giovannoni G. Multiple sclerosis - a review. *Eur J Neurol*. 2019;26:27-40.
25. Adelman G, Rane SG, Villa KF. The cost burden of multiple sclerosis in the United States: a systematic review of the literature. *J Med Econ*. 2013;16:639-647.
26. Chruzander C, Johansson S, Gottberg K, Einarsson U, Fredrikson S, Holmqvist LW, et al. A 10-year follow-up of a population-based study of people with multiple sclerosis in Stockholm, Sweden: changes in disability and the value of different factors in predicting disability and mortality. *J Neurol Sci*. 2013;332:121-127.
27. Bethoux F. Gait disorders in multiple sclerosis. *Continuum (Minneap Minn)*. 2013;19:1007-22.
28. Jørgensen M, Dalgas U, Wens I, Hvid LG. Muscle strength and power in persons with multiple sclerosis - A systematic review and meta-analysis. *J Neurol Sci*. 2017;376:225-241.
29. Fisk JD, Pontefract A, Ritvo PG, Archibald CJ, Murray TJ. The impact of fatigue on patients with multiple sclerosis. *Can J Neurol Sci*. 1994;21:9-14.
30. Timkova V, Mikula P, Fedicova M, Szilasiova J, Nagyova I. Psychological well-being in people with multiple sclerosis and its association with illness perception and self-esteem. *Mult Scler Relat Disord*. 2021;54:103114.
31. Chiaravalloti ND, DeLuca J. Cognitive impairment in multiple sclerosis. *Lancet Neurol*. 2008;7:1139-1151.
32. Coote S, Finlayson M, Sosnoff JJ. Level of mobility limitations and falls status in persons with multiple sclerosis. *Arch Phys Med Rehabil*. 2014;95:862-866.
33. Hayes S, Galvin R, Kennedy C, Finlayson M, McGuigan C, Walsh CD, et al. Interventions for preventing falls in people with multiple sclerosis. *Cochrane Database Syst Rev*. 2019;11:CD012475.
34. Kobelt G, Thompson A, Berg J, Gannedahl M, Eriksson J; MSCOI Study Group; European Multiple Sclerosis Platform. New insights into the burden and costs of multiple sclerosis in Europe. *Mult Scler*. 2017;23:1123-1136.
35. McGinley MP, Goldschmidt CH, Rae-Grant AD. Diagnosis and Treatment of Multiple Sclerosis: A Review. *JAMA*. 2021;325:765-779.

36. Motl RW, Mowry EM, Ehde DM, LaRocca NG, Smith KE, Costello K, et al. Wellness and multiple sclerosis: The National MS Society establishes a Wellness Research Working Group and research priorities. *Mult Scler*. 2018;24:262-267.
37. Proietti M, Cesari M. Frailty: What Is It? *Adv Exp Med Biol*. 2020;1216:1-7.
38. Hoogendijk EO, Afilalo J, Ensrud KE, Kowal P, Onder G, Fried LP. Frailty: implications for clinical practice and public health. *Lancet*. 2019;394:1365-1375.
39. Yarnall AJ, Sayer AA, Clegg A, Rockwood K, Parker S, Hindle JV. New horizons in multimorbidity in older adults. *Age Ageing*. 2017;46:882-888.
40. Dent E, Martin FC, Bergman H, Woo J, Romero-Ortuno R, Walston JD. Management of frailty: opportunities, challenges, and future directions. *Lancet*. 2019;394:1376-1386.
41. Zazzara MB, Vetrano DL, Carfì A, Onder G. Frailty and chronic disease. *Panminerva Med*. 2019;61:486-492.
42. Zanotto T, Rice LA, Sosnoff JJ. Frailty among people with multiple sclerosis who are wheelchair users. *PLoS One*. 2022;17:e0271688.
43. Zanotto T, Galperin I, Mirelman A, Chen L, Regev K, Karni A, et al. Association between frailty and free-living walking performance in people with multiple sclerosis. *Phys. Ther* (Under Review - 2022).
44. Friese MA, Schattling B, Fugger L. Mechanisms of neurodegeneration and axonal dysfunction in multiple sclerosis. *Nat Rev Neurol*. 2014;10:225-238.
45. Franceschi C, Campisi J. Chronic inflammation (inflammaging) and its potential contribution to age-associated diseases. *J Gerontol A Biol Sci Med Sci*. 2014;69:S4-9.
46. Ferrucci L, Fabbri E. Inflammageing: chronic inflammation in ageing, cardiovascular disease, and frailty. *Nat Rev Cardiol*. 2018;15:505-522.
47. Fairhall N, Kurrle SE, Sherrington C, Lord SR, Lockwood K, John B, et al. Effectiveness of a multifactorial intervention on preventing development of frailty in pre-frail older people: study protocol for a randomised controlled trial. *BMJ Open*. 2015;5:e007091.
48. Angulo J, El Assar M, Álvarez-Bustos A, Rodríguez-Mañas L. Physical activity and exercise: Strategies to manage frailty. *Redox Biol*. 2020;35:101513.
49. Puts MTE, Toubasi S, Andrew MK, Ashe MC, Ploeg J, Atkinson E, et al. Interventions to prevent or reduce the level of frailty in community-dwelling older adults: a scoping review of the literature and international policies. *Age Ageing*. 2017;46:383-392.
50. Apóstolo J, Cooke R, Bobrowicz-Campos E, Santana S, Marcucci M, Cano A, et al. Effectiveness of interventions to prevent pre-frailty and frailty progression in older adults: a systematic review. *JBI Database System Rev Implement Rep*. 2018;16:140-232.
51. Cadore EL, Sáez de Asteasu ML, Izquierdo M. Multicomponent exercise and the hallmarks of frailty: Considerations on cognitive impairment and acute hospitalization. *Exp Gerontol*. 2019;122:10-14.
52. Amatya B, Khan F, Galea M. Effectiveness of rehabilitation interventions for people with multiple sclerosis - A Cochrane Review summary with commentary. *NeuroRehabilitation*. 2019;45:429-431.
53. Taul-Madsen L, Connolly L, Dennett R, Freeman J, Dalgas U, Hvid LG. Is Aerobic or Resistance Training the Most Effective Exercise Modality for Improving Lower Extremity Physical Function and Perceived Fatigue in People With Multiple Sclerosis? A Systematic Review and Meta-analysis. *Arch Phys Med Rehabil*. 2021;102:2032-2048.
54. Andrade LEL, New York BSAC, Gonçalves RSDSA, Fernandes SGG, Maciel ÁCC. Mapping instruments for assessing and stratifying frailty among community-dwelling older people: a scoping review. *BMJ Open*. 2021;11:e052301.
55. Searle SD, Mitnitski A, Gahbauer EA, Gill TM, Rockwood K. A standard procedure for creating a frailty index. *BMC Geriatr*. 2008;8:24.
56. Martin FC, O'Halloran AM. Tools for Assessing Frailty in Older People: General Concepts. *Adv Exp Med Biol*. 2020;1216:9-19.

57. Zanotto T, Lynch SG, Hausdorff JM, Sosnoff JJ. Frailty in multiple sclerosis: A closer look at the deficit accumulation framework. *Mult Scler*. 2022;28:1000-1001.
58. Ghasemi N, Razavi S, Nikzad E. Multiple Sclerosis: Pathogenesis, Symptoms, Diagnoses and Cell-Based Therapy. *Cell J*. 2017;19:1-10.
59. Moher D, Hopewell S, Schulz KF, Montori V, Gøtzsche PC, Devereaux PJ, et al. CONSORT 2010 explanation and elaboration: updated guidelines for reporting parallel group randomised trials. *BMJ*. 2010;340:c869.
60. Schulz KF, Altman DG, Moher D; CONSORT Group. CONSORT 2010 statement: updated guidelines for reporting parallel group randomised trials. *BMJ*. 2010;340:c332.
61. Kurtzke JF. Rating neurologic impairment in multiple sclerosis: an expanded disability status scale (EDSS). *Neurology*. 1983;33:1444-1452.
62. Kurtzke JF. On the origin of EDSS. *Mult Scler Relat Disord*. 2015;4:95-103.
63. Tombaugh TN, McIntyre NJ. The mini-mental state examination: a comprehensive review. *J Am Geriatr Soc*. 1992;40:922-935.
64. Zigmond AS, Snaith RP. The hospital anxiety and depression scale. *Acta Psychiatr Scand*. 1983;67:361-70.
65. Larson RD, Larson DJ, Baumgartner TB, White LJ. Repeatability of the timed 25-foot walk test for individuals with multiple sclerosis. *Clin Rehabil*. 2013;27:719-23.
66. Bohannon RW. Reference values for the five-repetition sit-to-stand test: a descriptive meta-analysis of data from elders. *Percept Mot Skills*. 2006;103:215-22.
67. Cronin J, Lawton T, Harris N, Kilding A, McMaster DT. A Brief Review of Handgrip Strength and Sport Performance. *J Strength Cond Res*. 2017;31:3187-3217.
68. Goldman MD, Marrie RA, Cohen JA. Evaluation of the six-minute walk in multiple sclerosis subjects and healthy controls. *Mult Scler*. 2008;14:383-90.
69. Sosnoff JJ, Weikert M, Dlugonski D, Smith DC, Motl RW. Quantifying gait impairment in multiple sclerosis using GAITRite technology. *Gait Posture*. 2011;34:145-7.
70. Hogrel JY, Benveniste O, Bachasson D. Routine monitoring of isometric knee extension strength in patients with muscle impairments using a new portable device: cross-validation against a standard isokinetic dynamometer. *Physiol Meas*. 2020;41:015003.
71. Baetge SJ, Filser M, Renner A, Ullrich S, Lassek C, Penner IK. On the validity of single tests, two-test combinations and the full Brief International Cognitive Assessment for Multiple Sclerosis (BICAMS) in detecting patients with cognitive impairment. *Mult Scler*. 2020;26:1919-1928.
72. Tombaugh TN. Trail Making Test A and B: normative data stratified by age and education. *Arch Clin Neuropsychol*. 2004;19:203-14.
73. Vickrey BG, Hays RD, Harooni R, Myers LW, Ellison GW. A health-related quality of life measure for multiple sclerosis. *Qual Life Res*. 1995;4:187-206.
74. Learmonth YC, Dlugonski D, Pilutti LA, Sandroff BM, Klaren R, Motl RW. Psychometric properties of the Fatigue Severity Scale and the Modified Fatigue Impact Scale. *J Neurol Sci*. 2013;331:102-107.
75. Severin R, Sabbahi A, Albarrati A, Phillips SA, Arena S. Blood Pressure Screening by Outpatient Physical Therapists: A Call to Action and Clinical Recommendations. *Phys Ther*. 2020;100:1008-1019.
76. Tzur I, Izhakian S, Gorelik O. Orthostatic hypotension: definition, classification and evaluation. *Blood Press*. 2019;28:146-156.
77. Heppner HJ, Bauer JM, Sieber CC, Bertsch T. Laboratory aspects relating to the detection and prevention of frailty. *Int J Prev Med*. 2010;1:149-157.
78. Teng M, Bensmail D, Hanachi M, Haddad R, Hugeron C, Lansaman T, et al. Nutritional status in patients with advanced-stage multiple sclerosis. *Eur J Neurol*. 2022;29:1730-1740.
79. Sorgun MH, Yucesan C, Tegin C. Is malnutrition a problem for multiple sclerosis patients? *J Clin Neurosci*. 2014;21:1603-1605.

80. Mulasi U, Kuchnia AJ, Cole AJ, Earthman CP. Bioimpedance at the bedside: current applications, limitations, and opportunities. *Nutr Clin Pract*. 2015;30:180-193.
81. Shema-Shiratzky S, Hillel I, Mirelman A, Regev K, Hsieh KL, Karni A, et al. A wearable sensor identifies alterations in community ambulation in multiple sclerosis: contributors to real-world gait quality and physical activity. *J Neurol*. 2020;267:1912-1921.
82. Jones DM, Song X, Rockwood K. Operationalizing a frailty index from a standardized comprehensive geriatric assessment. *J Am Geriatr Soc*. 2004;52:1929-33.
83. Orsmond GI, Cohn ES. The Distinctive Features of a Feasibility Study: Objectives and Guiding Questions. *OTJR (Thorofare N J)*. 2015;35:169-177.
84. Childs JD, Piva SR, Fritz JM. Responsiveness of the numeric pain rating scale in patients with low back pain. *Spine (Phila Pa 1976)*. 2005;30:1331-1334.
85. Borg GA. Psychophysical bases of perceived exertion. *Med Sci Sports Exerc*. 1982;14:377-381.
86. Morishita S, Tsubaki A, Nakamura M, Nashimoto S, Fu JB, Onishi H. Rating of perceived exertion on resistance training in elderly subjects. *Expert Rev Cardiovasc Ther*. 2019;17:135-142.
87. Sosnoff JJ, Weikert M, Dlugonski D, Smith DC, Motl RW. Quantifying gait impairment in multiple sclerosis using GAITRite technology. *Gait Posture*. 2011;34:145-7.
88. Vieira JG, Sardeli AV, Dias MR, Filho JE, Campos Y, Sant'Ana L, et al. Effects of Resistance Training to Muscle Failure on Acute Fatigue: A Systematic Review and Meta-Analysis. *Sports Med*. 2022;52:1103-1125.
89. Izquierdo M, Ibañez J, Calbet JA, González-Izal M, Navarro-Amézqueta I, Granados C, et al. Neuromuscular fatigue after resistance training. *Int J Sports Med*. 2009;30:614-623.
90. Braith RW, Graves JE, Leggett SH, Pollock ML. Effect of training on the relationship between maximal and submaximal strength. *Med Sci Sports Exerc*. 1993;25:132-138.
91. International Conference on Harmonization. Clinical safety data management: definitions and standards for expedited reporting E2A. http://www.ich.org/products/guidelines/efficacy/efficacy-single/article/clinical-safety-data-management-definitions-and-standards-for-expedited-reporting.html
92. Morley JE, Malmstrom TK, Miller DK. A simple frailty questionnaire (FRAIL) predicts outcomes in middle aged African Americans. J Nutr Health Aging. 2012 Jul;16(7):601-8. doi: 10.1007/s12603-012-0084-2.
93. de Vries NM, Staal JB, Olde Rikkert MG, Nijhuis-van der Sanden MW. Evaluative frailty index for physical activity (EFIP): a reliable and valid instrument to measure changes in level of frailty. Phys Ther. 2013 Apr;93(4):551-61. doi: 10.2522/ptj.20120127.
94. Lord SR, Menz HB, Tiedemann A. A physiological profile approach to falls risk assessment and prevention. Phys Ther. 2003 Mar;83(3):237-52. PMID: 12620088.
95. Freynhagen R, Tölle TR, Gockel U, Baron R. The painDETECT project – far more than a screening tool on neuropathic pain. Current Medical Research and Opinion. 2016/06/02 2016;32(6):1033-1057. doi:10.1185/03007995.2016.1157460
96. Lacourt TE, Houtveen JH, Doornen LJPv. Experimental pressure-pain assessments: Test-retest reliability, convergence and dimensionality. Scandinavian Journal of Pain. 2012;3(1):31-37. doi:doi:10.1016/j.sjpain.2011.10.003

<u>APPENDIX I: VULNERABLE POPULATIONS</u>

I. Vulnerable populations are not being recruited.

II.
