# Supplementary material for: Reducing frailty in frail people with multiple sclerosis: Feasibility of a 6-week multimodal exercise training program
Source: PLoS One. 2026 Apr 15;21(4):e0347063. doi: 10.1371/journal.pone.0347063 (PMC13082602; doi:10.1371/journal.pone.0347063)
Supplement: S3 IRB approval — (PDF) [file pone.0347063.s003.pdf]

The University of Kansas Medical Center

Human Research Protection Program

APPROVAL OF SUBMISSION

July 6, 2023

Tobia Zanotto
tzanotto@kumc.edu

Dear Tobia Zanotto:

On 7/6/2023, the IRB reviewed the following submission:

Type of Review:	Initial Study
Reviewing IRB:	IRB00000161
FWA#:	00003411
IRB#:	STUDY00149742
Title:	Multimodal Exercise Training to Reduce Frailty in People with Multiple Sclerosis
Investigator:	Tobia Zanotto
IRB ID:	STUDY00149742
Funding:	None
IND, IDE or HDE:	None
Documents submitted for the above review:	• Borg scale<br>• BVMT questionnaire<br>• CVLT-II questionnaire<br>• frail scale<br>• MFIS questionnaire<br>• Mini-cog questionnaire<br>• MSQoL-54 questionnaire<br>• OMNI scale<br>• Informed Consent clean version<br>• Pain NRS scale<br>• DSMB Charter<br>• SDMT questionnaire<br>• Principal Investigator Supplement<br>• SPFQ questionnaire

	• IRB Checklist<br>• TMT questionnaire<br>• Scientific Review<br>• Study protocol<br>• Request for single IRB<br>• Informed Consent Form
Special Determinations:	None

The IRB approved this submission from 7/6/2023 to 1/5/2024 inclusive.

Your approved documents are stored in the “Documents” tab for this study in the eCompliance system. The IRB stamped consent form(s) can be found under the “Final” column on the right side of the screen. These are the **only** valid versions for documenting informed consent.

If continuing review approval is not granted on or before 1/5/2024, approval of this study expires after that date.

Approval of this research is contingent upon your agreement to:

(1) Adhere to all KUMC Policies and Procedures Relating to Human Subjects, as written in accordance with the Code of Federal Regulations (45 CFR 46).

(2) Ensure that all study personnel are adequately trained for their role on the study.

(3) Maintain current training in human subjects protection and current disclosure of conflicts of interest as required by KUMC policy.

(4) Except where informed consent and HIPAA authorization have been formally waived by the IRB, seek, document and maintain records of informed consent and HIPAA authorization from each prospective subject or his/her legally authorized representative.

(5) Maintain copies of all pertinent information related to the research study including, but not limited to, video and audio tapes, instruments, copies of written informed consent agreements, and any other supportive documents in accordance with the KUMC Research Records Retention Policy.

(6) Report adverse events, non-compliance and other problems to the IRB by submitting a Report of New Information.

(7) Follow the IRB-approved protocol. Submit Modifications to the IRB for any proposed changes from the previously approved project. Changes may not be initiated without prior IRB review and approval, unless a delay in implementation would place subjects at risk.

(8) Submit a Continuing Review to the KUMC IRB before the expiration date. Federal regulations and IRB policies require continuing review of research at intervals appropriate to the degree of risk, but not less than once per year.

For more information on Human Subjects Research Policies or using the eCompliance system, please see our website at: https://www.kumc.edu/research/research-administration/institutional-review-board.html.

If you have any questions regarding the human subject protection process, please do not hesitate to contact our office at 913-588-1240 or IRBhelp@kumc.edu.

Sincerely,

Michael Risse

Human Research Protection Program

APPROVAL

February 23, 2024

Tobia Zanotto
3901 Rainbow Blvd.
Kansas City, KS 66160

tzanotto@kumc.edu

Dear Tobia Zanotto:

Type of Review:	Modification / Update
Title:	Multimodal Exercise Training to Reduce Frailty in People with Multiple Sclerosis
Investigator:	Tobia Zanotto
IRB ID:	MOD00060354
STUDY ID:	STUDY00149742
Expedited/Exempt Category:	Expedited
Documents Reviewed:	• Protocol-ERFMS_02162024_proposed_amendments_tracked_<br>• version.doc<br>• Protocol-ERFMS_02162024_proposed_amendments_clean_<br>• version.doc<br>• Reconsenting plan<br>• Study00149742_02162024_proposed_amendments_clean_<br>• version.docx<br>• Study00149742_02162024_proposed_amendments_tracked_<br>• version.docx<br>• PainDETECT.pdf<br>• FM score.pdf

The IRB approved the protocol from 2/22/2024 to 6/18/2024 inclusive. Before 6/18/2024 or within 30 days of study close, whichever is earlier, you are to submit a completed continuing review and required attachments to request continuing appro

val or closure.

If continuing review approval is not granted before the expiration date of 6/18/2024, approval of this protocol expires on that date.

Your approved documents are stored in the "Documents" tab for this study in the eCompliance system. The IRB stamped consent form(s) can be found under the "Final" column on the right side of the screen. These are the only valid versions for documenting informed consent.

If continuing review approval is not granted on or before end date listed above, approval of this study expires after that date.

Approval of this research is contingent upon your agreement to:

(1) Adhere to all KUMC Policies and Procedures Relating to Human Subjects, as written in accordance with the Code of Federal Regulations (45 CFR 46).

(2) Ensure that all study personnel are adequately trained for their role on the study.

(3) Maintain current training in human subjects protection and current disclosure of conflicts of interest as required by KUMC policy.

(4) Except where informed consent and HIPAA authorization have been formally waived by the IRB, seek, document and maintain records of informed consent and HIPAA authorization from each prospective subject or his/her legally authorized representative.

(5) Maintain copies of all pertinent information related to the research study including, but not limited to, video and audio tapes, instruments, copies of written informed consent agreements, and any other supportive documents in accordance with the KUMC Research Records Retention Policy.

(6) Report adverse events, non-compliance and other problems to the IRB by submitting a Report of New Information.

(7) Follow the IRB-approved protocol. Submit Modifications to the IRB for any proposed changes from the previously approved project. Changes may not be initiated without prior IRB review and approval, unless a delay in implementation would place subjects at risk.

(8) Submit a Continuing Review to the KUMC IRB before the expiration date. Federal regulations and IRB policies require continuing review of research at intervals appropriate to the degree of risk, but not less than once per year.

For more information on Human Subjects Research Policies or using the eCompliance system, please see our website at: https://www.kumc.edu/research/research-administration/institutional-review-board.html.

If you have any questions regarding the human subject protection process, please do not hesitate to contact our office at 913-588-1240 or IRBhelp@kumc.edu.

Sincerely,

Jane Salisbury

Human Research Protection Program

APPROVAL

June 18, 2024

Tobia Zanotto
3901 Rainbow Blvd.
Kansas City, KS 66160

tzanotto@kumc.edu

Dear Tobia Zanotto:

Type of Review:	Continuing Review
Title:	Multimodal Exercise Training to Reduce Frailty in People with Multiple Sclerosis
Investigator:	Tobia Zanotto
IRB ID:	CR00020413
STUDY ID:	STUDY00149742
Reviewing IRB:	IRB00000161
Funding:	None
IND, IDE, or HDE:	None
Documents Reviewed:	• Protocol-ERFMS_02162024_proposed_amendments_clean_version.doc<br>• Study00149742_02162024_proposed_amendments_clean_version.pdf<br>• Continuing Review Supplement<br>• DSMB report signed by chairperson<br>• Letter with further explanations from PI

The IRB approved the protocol from 6/18/2024 to 6/17/2025 inclusive. Before 6/17/2025 or within 30 days of study close, whichever is earlier, you are to submit a completed continuing review and required attachments to request continuing approval or closure.

If continuing review approval is not granted before the expiration date of 6/17/2025, approval of this protocol expires on that date.

Your approved documents are stored in the “Documents” tab for this study in the eCompliance system. The IRB stamped consent form(s) can be found under the “Final” column on the right side of the screen. These are the only valid versions for documenting informed consent.

If continuing review approval is not granted on or before end date listed above, approval of this study expires after that date.

Approval of this research is contingent upon your agreement to:

(1) Adhere to all KUMC Policies and Procedures Relating to Human Subjects, as written in accordance with the Code of Federal Regulations (45 CFR 46).

(2) Ensure that all study personnel are adequately trained for their role on the study.

(3) Maintain current training in human subjects protection and current disclosure of conflicts of interest as required by KUMC policy.

(4) Except where informed consent and HIPAA authorization have been formally waived by the IRB, seek, document and maintain records of informed consent and HIPAA authorization from each prospective subject or his/her legally authorized representative.

(5) Maintain copies of all pertinent information related to the research study including, but not limited to, video and audio tapes, instruments, copies of written informed consent agreements, and any other supportive documents in accordance with the KUMC Research Records Retention Policy.

(6) Report adverse events, non-compliance and other problems to the IRB by submitting a Report of New Information.

(7) Follow the IRB-approved protocol. Submit Modifications to the IRB for any proposed changes from the previously approved project. Changes may not be initiated without prior IRB review and approval, unless a delay in implementation would place subjects at risk.

(8) Submit a Continuing Review to the KUMC IRB before the expiration date. Federal regulations and IRB policies require continuing review of research at intervals appropriate to the degree of risk, but not less than once per year.

For more information on Human Subjects Research Policies or using the eCompliance system, please see our website at: https://www.kumc.edu/research/research-administration/institutional-review-board.html.

If you have any questions regarding the human subject protection process, please do not hesitate to contact our office at 913-588-1240 or IRBhelp@kumc.edu.

Sincerely,

Jane Salisbury

Human Research Protection Program

CLOSURE

June 16, 2025

Tobia Zanotto
3901 Rainbow Blvd.
Kansas City, KS 66160-8500

tzanotto@kumc.edu

Dear Tobia Zanotto:

On 6/16/2025, the IRB reviewed the following submission:

Type of Review:	Continuing Review
Title:	Multimodal Exercise Training to Reduce Frailty in People with Multiple Sclerosis
Investigator:	Tobia Zanotto
IRB ID:	CR00021018
STUDY ID:	STUDY00149742

The IRB acknowledges your request for closure of the protocol effective as of 6/16/2025. As part of this action:

- The protocol is permanently closed to enrollment.
- All subjects have completed all protocol-related interventions.
- Collection of private identifiable information is completed.
- Analysis of private identifiable information is completed.

Sincerely,
Morgan Martin-West
